# Structural hot spots for the solubility of globular proteins

**DOI:** 10.1038/ncomms10816

**Published:** 2016-02-24

**Authors:** Ashok Ganesan, Aleksandra Siekierska, Jacinte Beerten, Marijke Brams, Joost Van Durme, Greet De Baets, Rob Van der Kant, Rodrigo Gallardo, Meine Ramakers, Tobias Langenberg, Hannah Wilkinson, Frederik De Smet, Chris Ulens, Frederic Rousseau, Joost Schymkowitz

**Affiliations:** 1 VIB Switch Laboratory, Flanders Institute for Biotechnology (VIB), 3000 Leuven, Belgium; 2 KU Leuven, Switch Laboratory, Department of Cellular and Molecular Medicine, Herestraat 49, PB 802, 3000 Leuven, Belgium; 3 Vrije Universiteit Brussel, Pleinlaan 2, 1050 Brussel, Belgium; 4 KU Leuven, Laboratory for Structural Neurobiology, Department of Cellular and Molecular Medicine, Herestraat 49, PB 601, B-3000 Leuven, Belgium

## Abstract

Natural selection shapes protein solubility to physiological requirements and recombinant applications that require higher protein concentrations are often problematic. This raises the question whether the solubility of natural protein sequences can be improved. We here show an anti-correlation between the number of aggregation prone regions (APRs) in a protein sequence and its solubility, suggesting that mutational suppression of APRs provides a simple strategy to increase protein solubility. We show that mutations at specific positions within a protein structure can act as APR suppressors without affecting protein stability. These hot spots for protein solubility are both structure and sequence dependent but can be computationally predicted. We demonstrate this by reducing the aggregation of human α-galactosidase and protective antigen of *Bacillus anthracis* through mutation. Our results indicate that many proteins possess hot spots allowing to adapt protein solubility independently of structure and function.

## Abstract (web summary)

Mutations in aggregation prone regions of recombinant proteins often improve their solubility, although they might cause negative effects on their structure and function. Here, the authors identify proteins hot spots that can be exploited to optimize solubility without compromising stability.

## Graphical abstract

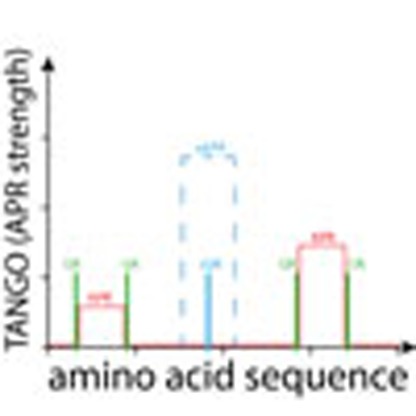

To function proteins have to fold into their native structure while avoiding misfolding and aggregates. However, the spread in cellular abundance of proteins in a typical proteome spans about nine to ten orders of magnitude^1^. As protein aggregation is a concentration-dependent process the challenge posed to protein folding therefore varies enormously between low- and high-abundant proteins^2^. Recent evidence indicates the aggregation propensity of proteins is tuned to their cellular abundance^3^,^4^ and that cellular protein levels correlate with protein solubility^5^. This suggests protein solubility is on average only marginally superior to physiological expression levels and that many proteins are ‘living on the edge'^6^.

This has important implications for the production and use of recombinant proteins in biotechnology and therapeutics. Recombinant protein applications require protein concentrations several orders of magnitude above natural abundance. As a result several potentially valuable therapeutic proteins remain beyond reach. This also raises fundamental questions on the evolutionary mechanisms allowing adapting protein solubility to changing requirements in protein abundance. Indeed, reducing the aggregation propensity of globular proteins is not a straightforward task. Globular structure requires a hydrophobic core to lock secondary structure elements into a well defined three-dimensional fold, which in turn generates aggregation prone amino acid sequences^7^,^8^. Protein structure and protein aggregation are therefore entangled properties, rendering it very difficult to remove protein aggregation without affecting protein stability and structure^9^. The question therefore remains how natural selection manages to co-evolve protein function and solubility without affecting the native structure of proteins and by extension, what we can learn from this to improve the properties of industrial and therapeutic recombinant proteins.

Protein aggregation is a process resulting in the accumulation of misfolded proteins into insoluble agglomerates. Although hydrophobicity is often an important driver for phase separation of misfolded proteins, structural aggregation itself is geared by more specific interactions between identical linear aggregation prone sequence regions (APRs) within the primary sequence that assemble by intermolecular β-strand interactions. The determining role of APRs in protein aggregation has been demonstrated for several amyloid-disease proteins by grafting experiments: insertion of APRs from various proteins in a non-aggregating scaffold domain results in similar aggregation propensity and morphology than the original protein from which the APR was derived^10^,^11^,^12^. On average a globular protein domain contains 2–4 APRs and ∼20% of the total protein sequence of any given proteome is part of APRs^13^,^14^. It was demonstrated on a large set of *Escherichia coli* proteins that the intrinsic aggregation propensity of folded proteins correlates with their solubility^15^.

We show the solubility of proteins can directly be related to the number of APRs within a protein sequence and that modulating the number of APRs by mutation significantly affects protein solubility. Moreover, we perform an analysis of the relationship between protein structure and the aggregation propensity of its primary sequence over a representative set of 584 high quality crystal structures (R-factor <0.19 and resolution<1.5 Å) (ref. 16), representing all common protein folds. We find that although most residues within APRs are in a structural gridlock coupling aggregation and thermodynamic stability, specific positions in a protein structure can be mutated to lower the aggregation propensity of the primary sequence without significantly affecting protein stability. These context-dependent hotspots for solubility therefore allow to significantly improve protein solubility in a step-wise manner by point mutations that suppress individual APRs. These findings further clarify our understanding of the selective pressures relating protein structure, function and solubility. It confirms that although APRs cannot be avoided altogether in globular proteins, protein aggregation is under selective pressure. Our structural analysis demonstrates this selective pressure is not saturated but dictated by physiological requirements in protein abundance and as a result most proteins have potential for increased solubility. We illustrate these principles with two examples in which we introduced mutations in these structural hotspots thereby increasing resistance to protein aggregation without affecting structure or function: α-galactosidase, a protein currently used in replacement therapy for Fabry's disease and the anthrax protective antigen, a key component for recombinant anthrax vaccines.

## Results

### Protein abundance correlates with aggregation prone regions

**Figure 1:**
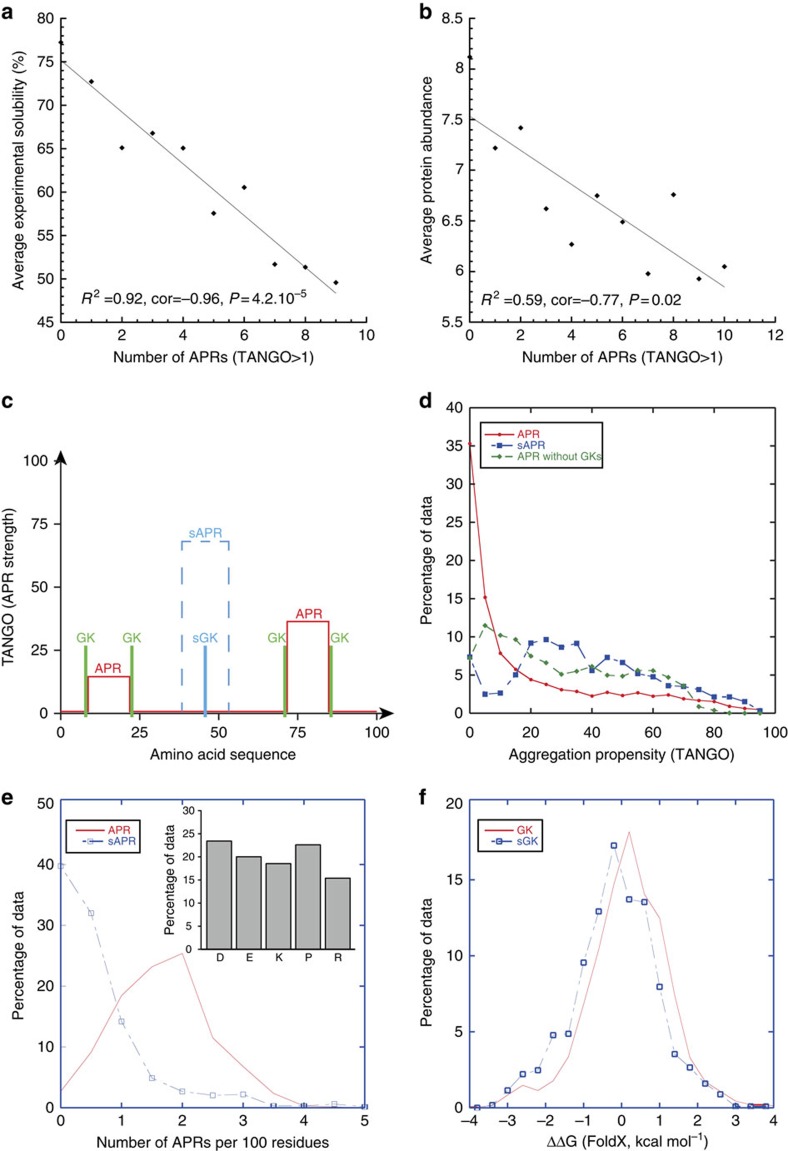
APRs and gatekeepers in natural polypeptide sequences. (**a**) The scatterplot of the average experimentally determined solubility^5^,^17^ of *E. coli* proteins grouped by the number of APRs in their sequence displays an anti-correlation, showing that on average protein solubility decreases with increasing number of aggregation prone sequences. (**b**) A similar plot to A, but now for protein abundance in *E. coli in vivo* 5,17. Although the anti-correlation is weaker, the trend suggests that cellular abundance is also limited by the number of APRs in its sequence. (**c**) Schematic representation depicting the TANGO profile of a typical naturally occurring polypeptide stretch of 100 amino acids, containing two APRs (red) each flanked by an N-terminal as well as a C-terminal gatekeeper (green), as well as one suppressed APR (blue) controlled by a single suppressing gatekeeper. (**d**) A density plot of the TANGO scores in the set of 634 structurally diverse proteins (red), showing a strong bias towards lower scores. In the absence of gatekeeper residues, this distribution is biased towards higher scores (green). The TANGO scores of the suppressed APRs (blue) show a more even distribution including higher scoring regions. (**e**) Density plot showing the number of APRs occurring per 100 amino acids in 634 structurally diverse proteins (red line). The blue line shows the number of ‘suppressed APRs', which are not detected by TANGO in the WT sequence, but show up after mutation of a single Asp, Glu, Lys, Arg or Pro residue to Ala. If mutation of such a residue reveals an APR that is undetectable in the WT sequence, we call that residue a ‘suppressing gatekeeper'. The inset shows the frequency of the amino acid type of suppressing gatekeepers. (**f**) Density plot of the contribution of gatekeeper residues to the overall thermodynamic stability of the native state of the protein as calculated by FoldX (red). The same plot for the suppressing gatekeepers in the same proteins (blue) is very similar.

We used TANGO to predict all APRs in the *E. coli* proteome and matched these (1) to *E. coli* protein solubility (3,173 proteins) as measured by Niwa *et al*.^17^ using *in vitro* translation and (2) to cellular abundance (597 proteins) data obtained by Vogel *et al*.^18^ using mass spectrometry (Supplementary Fig. 1A). We find a good correlation (cor=−0.96) between the number of APRs and the protein solubility averaged by APR count. (Fig. 1a), consistent with the notion that the burden of additional APRs lowers the solubility of proteins. There is a large variation per category (Supplementary Fig. 1B), indicating the effect of APRs is modulated strongly by cellular and structural context. To ensure the correlation is robust, we performed a bootstrap analysis, in which we recalculated the same correlation based on 1,000 randomly selected samples, each consisting of 80% of the original data points. The bootstrapped correlations are consistently high (Supplementary Fig. 1C), supporting the robustness of the observation. Similarly, we find a good correlation (cor=−0.77) between the number of APRs in a protein sequence and cellular abundance (Fig. 1b). Again, there is quite some variation per category (Supplementary Fig. 1D), but the correlation is robust in 1,000-fold bootstrap analysis (Supplementary Fig. 1E). It was previously shown that protein solubility *in vitro* and cellular protein abundance *in vivo* correlate, indicating that protein solubility is tuned to their cellular abundance^5^,^7^. Our results therefore suggest that altering the number of APRs would have a strong effect on protein solubility and abundance. Interestingly, the correlation between intrinsic aggregation propensity and protein abundance improves when a higher TANGO cutoff is employed (cor=−0.97), although with a reduced number of datapoints in the plot (Supplementary Fig. 1F), Nevertheless, this suggests that the stronger APRs are relevant for protein abundance *in vivo*, while all regions contribute to solubility *in vitro*.

### Protein sequences possess cryptic APRs

The existence of APRs is a consequence of globular structure and especially of the requirement of hydrophobic cores. At the same time it hampers protein folding and limits protein solubility. Not surprisingly it was found that APRs are subjected to negative selection^19^. The most efficient strategy to eliminate APRs consists in mutating a central hydrophobic residue in the APR to a charged residue or a Pro, as these so-called aggregation gatekeepers disfavour the β-structure adopted by hydrophobic sequences in aggregates^20^. However, in practice in most cases it is impossible to place charged residues at central positions within the APR without obliterating the native structure given that APRs are often part of the hydrophobic core. The next best solution to reduce aggregation is to place gatekeepers at the flanks of APRs (Fig. 1c) as these positions are relatively more solvent exposed allowing to accommodate charges with an acceptable cost to the thermodynamic stability of the protein^21^. Although this does generally not allow to completely suppress protein aggregation, the almost systematic placement of charges at the flanks of APRs clearly minimizes aggregation as can be observed from the exponential distribution of APR strength in the human proteome (Fig. 1d) with the majority of APRs displaying low-to-moderate aggregation tendencies. The green curve in the plot shows that this distribution in the absence of the gatekeeper residues is shifted towards much stronger aggregation propensities.

This general situation does not imply that it is necessarily always impossible to silence an APR by placement of a central gatekeeper. In fact the selective pressure against aggregation suggests that proteins should possess a significant amount of suppressed APRs (Fig. 1c). To investigate the existence of suppressed APRs we analysed a non-redundant set (sequence identity below 30%) of 584 proteins for which high quality crystal structures were available (R-factor better than 0.19, resolution better than 1.5 Å), which were previously selected by the WHATIF consortium^22^. First, we used the TANGO software^13^ to identify all APRs and GKs in these proteins. However when a gatekeeper residue is most effective, it will result in a sequence that no longer scores as aggregation prone and hence, these ‘suppressing gatekeepers' (sGKs) are not identified during the standard analysis because the APR will not be detected (it is suppressed). When such a residue is mutated to an amino acid that does not oppose aggregation, the aggregation score for the comprising region will dramatically increase, thus revealing the suppressed APR (Fig. 1c). Of course, unequivocally identifying suppressed APRs in this manner is impossible as we cannot make the difference between a previously existent APR that has been suppressed or a non-aggregating sequence that evolved one mutation away towards aggregation for other functional reasons. What this analysis can provide, however, is an idea of the number of cryptic APRs in a protein sequence and thus the number of positions within a sequence that are hotspots for susceptibility to aggregation upon mutation.

To identify sGKs we truncated all Arg, Lys, Glu, Asp and Pro residues outside of already identified APRs individually to Ala and employed TANGO to identify those residues whose truncation to Ala leads to the detection of a novel APR in the mutated sequence. The APR discovered in this way we call ‘cryptic APR' and the residue that keeps its aggregation propensity low in the wild-type (WT) sequence ‘suppressing GK'. It should be noted that the choice for mutating to Ala is guided by the structural considerations under study, that is, the interdependence of protein aggregation and stability. This conservative mutation is a good reporter both for aggregation (its aggregation propensity is a good approximation for the average of all amino acids) and for the thermodynamic contribution of that residue to the structure (mutation to Ala removes interactions without introducing new ones).

This analysis revealed that on average for every two regular APRs, proteins contain another cryptic APR and that 40% of proteins in this set have no sAPR, whereas this is less than 5% for real APRs (Fig. 1e) suggesting that indeed it is structurally more difficult to silence an APR by sGK than to cap the flanks of APRs with GK. The frequency of each of the five amino acid types of gatekeepers is roughly equal (Fig. 1e, inset) and in contrast to regular APRs, whose intrinsic aggregation propensity is strongly biased to the lowest TANGO scores, the cryptic APRs have TANGO scores across the entire spectrum (Fig. 1d). Taken together, these data suggest that any gatekeeper residue can be employed to silence the intrinsic aggregation propensity of an APR, and the limitation to its placement will come predominantly from compatibility with the folded structure. In order to evaluate this, we employed the FoldX algorithm^23^ to quantify the contribution of individuals GKs and sGKs to protein stability, which yielded similar distributions largely centred around zero (Fig. 1f). Importantly, the FoldX analysis suggests that ∼40% of GKs and sGKs come at a thermodynamic cost to the stability of the native structure, suggesting that the net gain for minimizing aggregation often outweigh the price paid in terms of thermodynamic stability.

### sGK mutations are enriched in disease mutants

**Figure 2:**
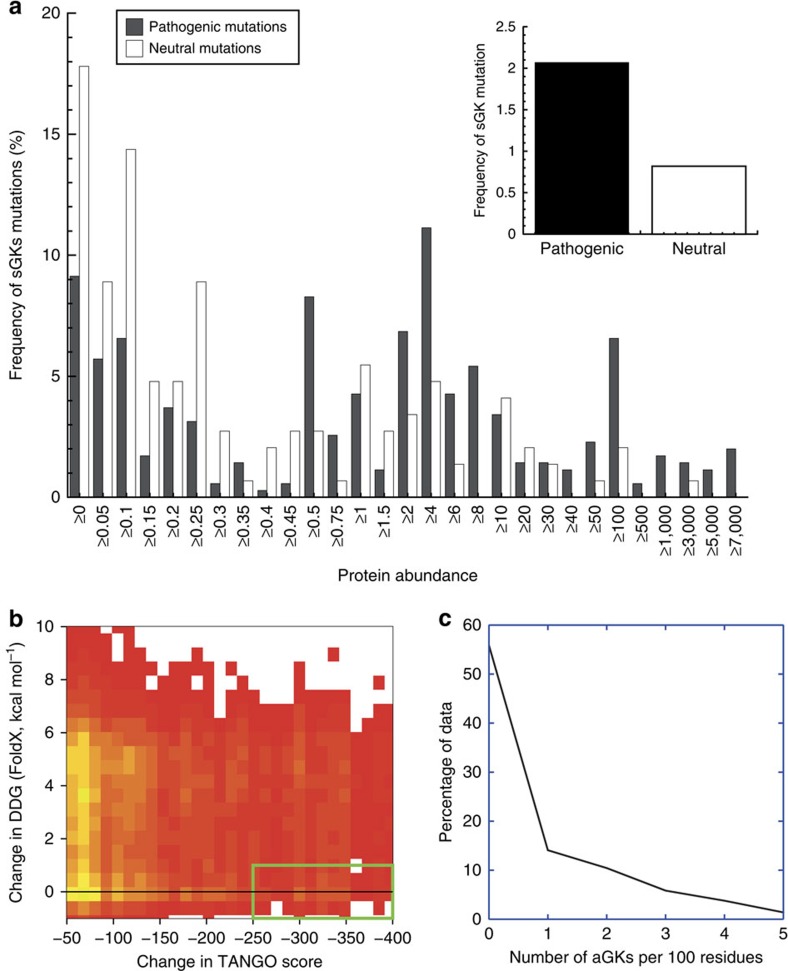
Potential for introducing artificial gatekeepers. (**a**) Comparison of the frequency of mutations that remove suppressing gakekeepers (sGKs) in pathogenic mutations and polymorphisms. The overall frequency is shown in the inset, whereas the main plot shows how the frequency of these mutations varies according to the cellular abundance of the WT proteins, showing that removal of a sGK is more likely to be neutral in proteins that occur at a low cellular concentration. (**b**) A systematic mutational scan of all residues of 4,730 APRs in 634 structurally diverse proteins. Each residue was mutated to Pro, Arg, Lys, Asp or Glu and its effect on intrinsic aggregation propensity and protein stability (ΔΔG) estimated using TANGO and FoldX, respectively. Heat map analysis (red to yellow gradient indicating increasing density) of the change in intrinsic aggregation propensity versus protein stability (ΔΔG) shows that the main density of mutations occurs at a high cost to thermodynamic stability and with moderate reductions of intrinsic aggregation and that only a minority of mutants significantly reduce aggregation without affecting thermodynamic stability (green box). (**c**) Density plot of the number of artificial gatekeepers within the green box of **b** that reduce significantly the aggregation propensity of an APR without affecting thermodynamic stability of the native structure (ΔTANGO<−250 and ΔΔG-value <1 kcal mol^−1^).

To further evaluate the importance of sGKs we analysed the frequency of loss of sGKs through mutation in the Varibench set of 19,335 disease mutations and 21,170 human non synonymous coding single-nucleotide polymorphisms. This revealed that the loss of a sGK is twice as likely in the disease set than in the polymorphism set (Fig. 2a, inset), suggesting that this type of mutation is likely to be deleterious, consistent with the notion that sGKs fulfil an important role in repressing cryptic APRs. In addition, we analysed PaxDB^24^, an integrated database for the natural abundance of human proteins, comparing the abundances of proteins that contain disease-associated and neutral mutations that affect sGKs (Fig. 2a). Statistical analysis shows these distributions differ significantly (Mann–Whitney U-test *P* value of 5.43 × 10^−11^), suggesting that mutation of a sGK in a highly abundant protein is more likely to be disease-associated than in a lowly abundant protein. This underlines the importance of this novel class of residues in polypeptide sequences.

### Decreasing aggregation without affecting structure

The overall selective tendency to minimize the aggregation propensity in a proteome by flanking gatekeepers or suppressing gatekeepers does not mean that all APRs are necessarily optimized to saturation point. This idea is compatible with the observation that protein solubility is generally optimized to its physiological abundance but generally not more^4^,^5^. The question therefore remains to what degree the aggregation of natural protein sequences is still amenable to improvement.

To investigate this, we systematically mutated every residue that is part of an APR in the same set of 634 proteins individually to Pro, Arg, Lys, Asp and Glu and recorded the predicted change in aggregation propensity (TANGO) and protein stability (ΔΔG). Figure 2b shows a density distribution of the resulting 75,720 mutations as a heat map from yellow (frequent) to red (rare), revealing as expected that for most positions in the protein sequence mutations improving the aggregation propensity of the sequence do so at a major cost for thermodynamic stability (values of 5 kcal mol^−1^are unlikely to occur in an experimental setting and result from strong Van der Waals clash penalties in FoldX). However, some density can be observed in the region of the plot corresponding to a strong reduction of intrinsic aggregation at a low cost to thermodynamic stability (<1 kcal mol^−1^), suggesting that additional gatekeepers at selected positions is structurally possible. As is shown in Fig. 2c, in about half the protein domains at least one such gatekeeper could be placed *in silico*, suggesting there is significant potential for protein improvement at specific sites within protein structures.

To validate these principles, we experimentally investigated the effect of introducing artificial gatekeeper mutations in two example proteins: the human protein α-galactosidase and protective antigen (PA) from *Bacillus anthracis*.

### Rational design of aggregation-resistant α-galactosidase

**Figure 3:**
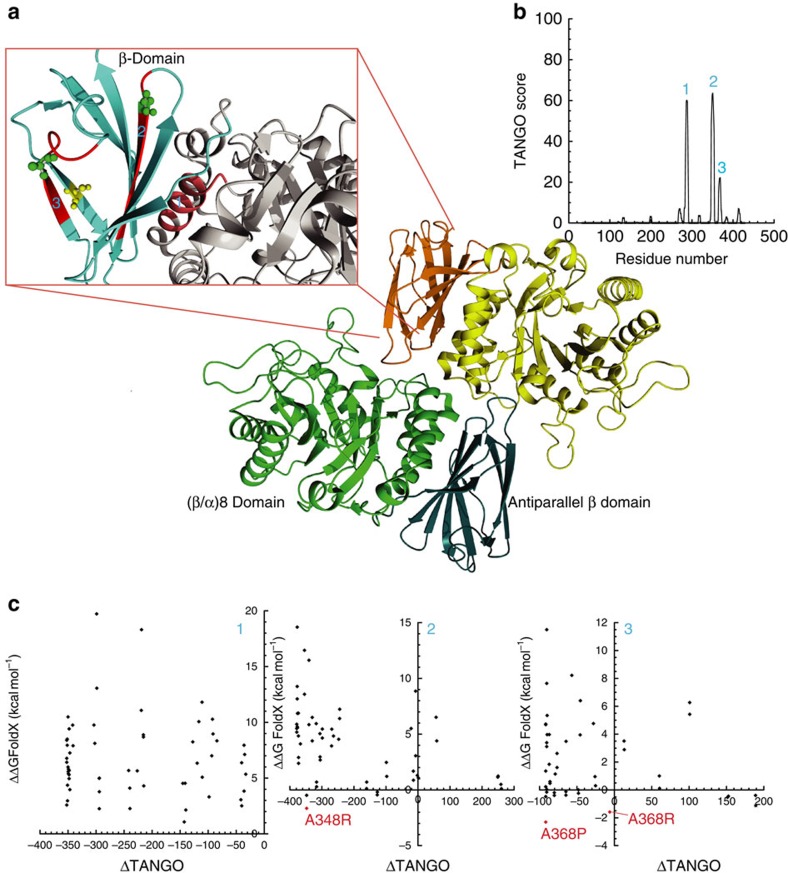
Structure and aggregation propensity of human WT α-Gal. (**a**) Human α-Gal is a homodimer (PDB 3GXP)^54^ in which each monomer contains a (β/α)8 domain (central part, yellow and green), where the active site is located and an antiparallel β-domain (central part, orange and blue). The structure was visualized with YASARA (ref. 53). (**b**) The intrinsic aggregation propensity of the α-Gal sequence as predicted by the TANGO algorithm reveals three strongly aggregation-prone regions: (1) M_284_ALWAIMA_291_; (2) L_347_AWAVAMI_355_; and (3) Y_365_TIAVAS_371_. The regions predicted by TANGO were indicated in the structure with numbers 1, 2 and 3 and were coloured in red (Fig. 1a). (**c**) Scatter plots representing the results of computational gatekeeper scans for each of the aggregation prone regions of α-Gal (ΔΔG FoldX versus ΔTANGO). For the TANGO region 2 mutation A348R could be identified (green amino acid residue in Fig. 1a), whereas for the TANGO region 3 mutations A368R and A368P (green amino acid residue in Fig. 1a) were identified.

Human α-galactosidase (α-Gal) is a lysosomal hydrolase. α-Gal deficiency results in Fabry disease (FD) (OMIM 301500), a metabolic X-linked inherited lysosomal storage disorder^25^,^26^. α-Galactosidase enzyme replacement therapy is currently used to treat FD. The structure of α-galactosidase consists of a homodimer, in which each monomer contains a (β/α) domain (Fig. 3a, central part, yellow and green parts) harbouring the active site and an antiparallel β-domain (Fig. 3a, orange and blue). The APRs of α-Gal (indicated with 1, 2 and 3 and coloured in red in Fig. 3b, upper left), cluster in the β-domain and the interface between the domains. From visual inspection of the structure, region 3 in particular is likely to be at risk of nucleating aggregation, given its edge position in the β-sheet. The results of the computational gatekeeper scan of each of the APRs of α-Gal are shown as a MASS-plot (mutant aggregation and stability spectrum), that is, a scatter plot (Fig. 3c) of the change in thermodynamic stability (ΔΔG values calculated by FoldX in kcal mol^−1^) versus change in the intrinsic aggregation propensity (values calculated by TANGO, range between 0 and 100 per amino acid residue) associated to each APR. These plots allow to easily identify ideal mutations with large negative values on both axes, that is, mutations that reduce the intrinsic aggregation propensity while not decreasing the thermodynamic stability of an APR. Confirming our bioinformatics analysis above, designable positions in α-Gal exist but are sparse and context-dependent. For example, for APR1, no improving mutations could be identified, owing mainly to its complete burial inside the tightly packed domain interface. APRs 2 and 3 display one favourable mutation each (A348R and A368P, respectively), with an additional mutation in APR3 (A368R) stabilizing the region without lowering intrinsic aggregation (Fig. 3c).

**Figure 4:**
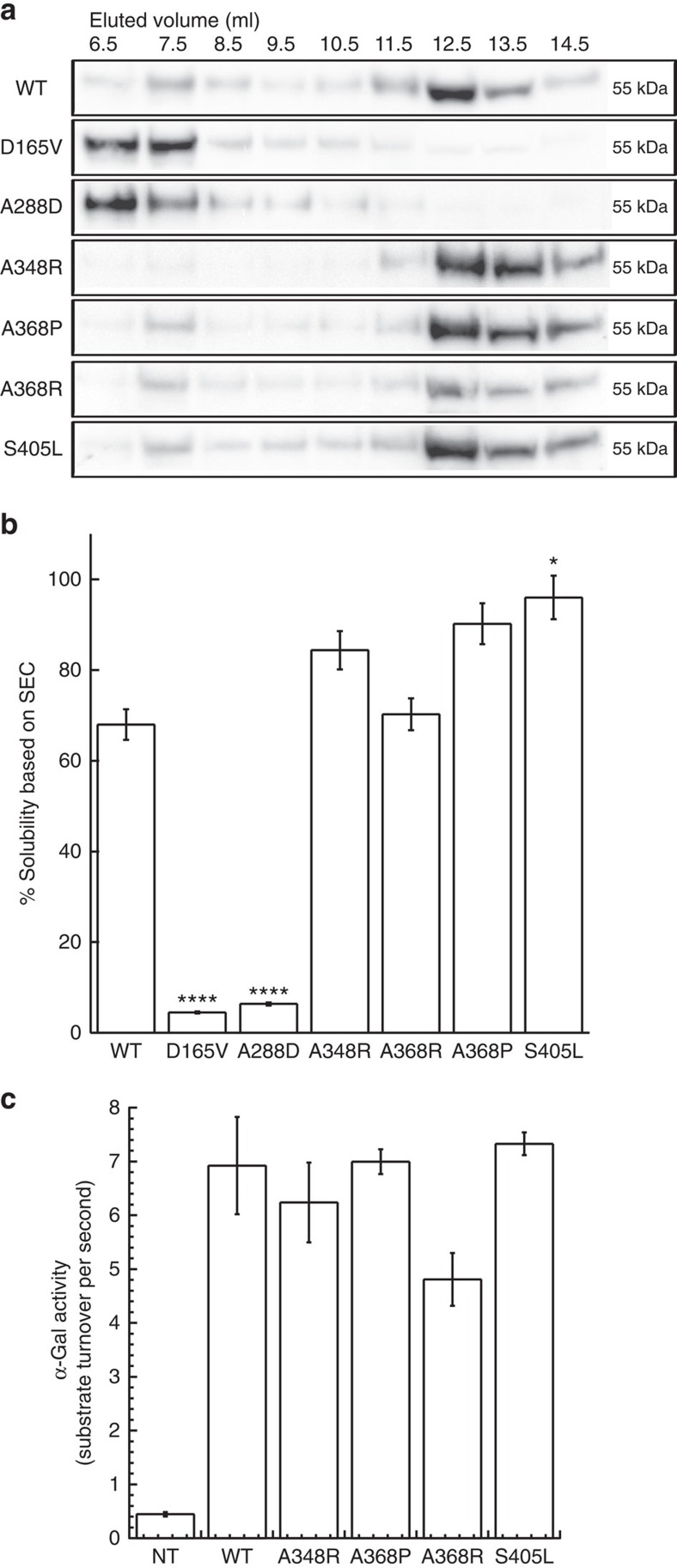
Effect of the single mutations on α-Gal aggregation and activity. (**a**) Western blot of SEC fractions of WT α-Gal, selected aggregating mutants and single mutants in transiently transfected HeLa cells. WT α-Gal and single mutants eluted in later fractions (12.5–14.5 ml) than the aggregating mutants, corresponding to the active soluble form of the protein. (**b**) Quantification of the solubility of α-Gal mutants in transiently transfected HeLa cells. The band densities from western blot of SEC fractions from several experiments were quantified. Fractions from 6.5–10.5 ml elution were considered as aggregated, whereas from 12.5–14.5 ml as monomeric. WT α-Gal showed approximately 75% solubility, whereas aggregating mutants were highly insoluble. Single mutants reached ∼80–90% of total solubility (with the exception of the A368R mutant). Statistical significance was calculated as compared with WT α-Gal; ‘*' *P*<0.05 and ‘****' *P*<0.0001. (**c**) Quantification of the enzymatic activity of WT α-Gal and single mutations in transiently transfected HeLa cells. The activity of the WT and the mutants was similar, with a slight reduction in the case of the A368R mutant. Values are mean±s.e.m.

In addition, to further reduce aggregation we also looked for mutations that stabilize the β-domain of the protein. Even though these do not reduce APRs, the additional thermodynamic stability will minimize the detrimental effect of introducing artificial gatekeepers at other sites. An exhaustive *in silico* mutation scan throughout the β-domain yielded a set of mutations increasing thermodynamic stability (Table 1). The most stabilizing mutation (S405L, ΔΔG_FoldX_=−3.34 kcal mol^−1^) tightens the interaction of the edge β-strand (the site of APR3) with the rest of the domain. We experimentally verified the effect of all mutations, comparing them with WT α-Gal and D165V and A288D, FD mutants previously found to be particularly aggregation prone^27^. Mutants were transiently overexpressed in HeLa cells and α-Gal solubility was measured in lysates by western blotting of size exclusion chromatography (SEC) elution fractions (material and methods). WT protein elutes mainly as a dimer but also shows faint bands in the SEC fractions eluting at volumes corresponding to large molecular sizes (Fig. 4a) (exclusion limit of this column is 600 kDa), consistent with partial misfolding and aggregation of WT α-Gal. As expected, the disease associated mutations D165V and A288D display significant increase of these high molecular weight fractions at the expense of the dimer fraction. The individual mutations selected to reduce aggregation show a modest decrease in the high molecular weight fractions when compared with WT α-Gal, that is most pronounced for the A348R mutant (Fig. 4a and Supplementary Fig. 2). Quantification of the solubility confirmed that while aggregating mutants (D165V and A288D) were highly insoluble (<10% of total protein), our single-point mutants reached ∼80–90% solubility compared with ∼70% for WT (Fig. 4b). We also determined the enzymatic activity of lysates of HEK293 cells transiently expressing α-Gal by following the conversion of the fluorogenic substrate 4-methylumbelliferyl-α-D-galactopyranoside (4-MU-α-Gal) over time. Single-point mutants showed similar enzymatic activity in comparison to WT (Fig. 4c), suggesting these mutations did not affect the active site of the enzyme and did not interfere with its enzymatic function. Overall, the effects of the single mutants are rather modest but show a decrease of aggregation and an improved solubility while leaving enzymatic activity unharmed. The fact that the observed improvements are overall modest is explained by the fact that α-Gal possesses three APRs: improving one by a single mutation leaves it susceptible to aggregation by the other regions. It is therefore expected that targeting several zones in parallel by multiple mutants should have a synergistic effect on the solubility of α-Gal.

**Figure 5:**
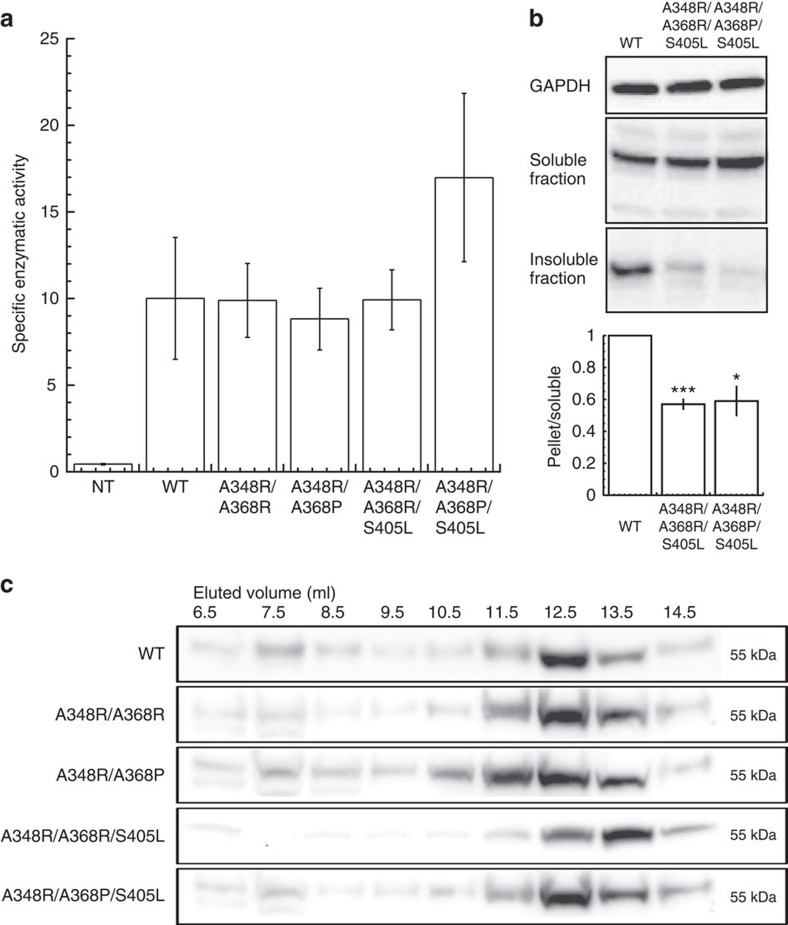
Effect of double and triple mutants on α-Gal aggregation and activity. (**a**) Quantification of the enzymatic activity of WT α-Gal and double and triple mutations in transiently transfected HeLa cells. The activity of the improved mutants was ∼twofold higher than the WT protein. Statistically significant values as compared with WT are indicated: ‘***' *P*< 0.001 and ‘****' *P*<0.0001. Data are means from three independent experiments; error bars show s.e.m. (**b**) Western blot of the level of WT α-Gal and mutants in the soluble and insoluble fraction of transiently transfected HeLa cells. The fraction of the mutants that is found in the insoluble fraction is significantly lower than for WT α-Gal (barplot, result of four repeats). (**c**) Western blot of SEC fractions of WT and double and triple mutants in transiently transfected HeLa cells. WT α-Gal and improving mutants eluted in later fractions (12.5–14.5 ml) corresponding to the active soluble form of the protein.

To determine the best combinations of mutations, we generated several double (A348R/A368P and A348R/A368R) and triple mutants (A348R/A368P/S405L and A348R/A368R/S405L) consisting of the single mutations in APRs 2and 3 and the stabilizing mutant S405L. First, we observed no significant changes in the intrinsic enzymatic activity of the mutants compared with WT (Fig. 5a) upon transient expression, showing our mutations had no major effects on the catalytic site. A similar level of soluble enzyme is reached (Fig. 5b) for WT and mutants, but there is a clear reduction of fraction of the mutants in the insoluble fraction by western blot (Fig. 5b and Supplementary Fig. 3). Moreover, this is mirrored by reduced accumulation of soluble aggregates for the mutants by SEC analysis (Fig. 5c and Supplementary Fig. 4). Together these data show the rational redesign of APRs in α-Gal is able to generate double and triple mutants that optimize protein solubility by reducing protein aggregation.

### Rational design of aggregation-resistant PA

**Figure 6:**
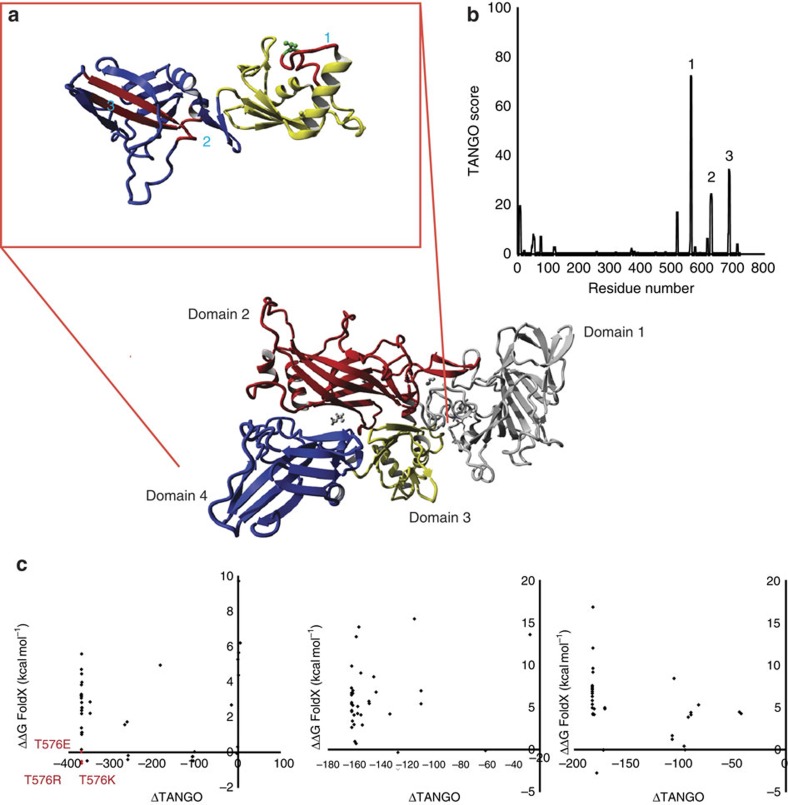
Structure and aggregation propensity of protective antigen (PA) of *B. anthracis*. (**a**) Monomeric PA is composed of four structural domains consisting predominantly of anti-parallel β-sheet: D1 is the calcium-binding domain (grey), D2 is the membrane insertion and heptamerization domain (red), D3 is the heptamerization domain (yellow) and D4 is the receptor binding domain (purple). (**b**) The intrinsic aggregation profile as predicted by TANGO indicated the presence of three main APRs, which are located in domains 3 and 4. The aggregation kinetics were shown to be dominated by the solvent exposed APR1 in the loop region of domain 3 (ref. 33). (**c**) MASS plots for the three APRs indicated in **b**. The mutations in APR 1 that we focused on are indicated in red.

**Figure 7:**
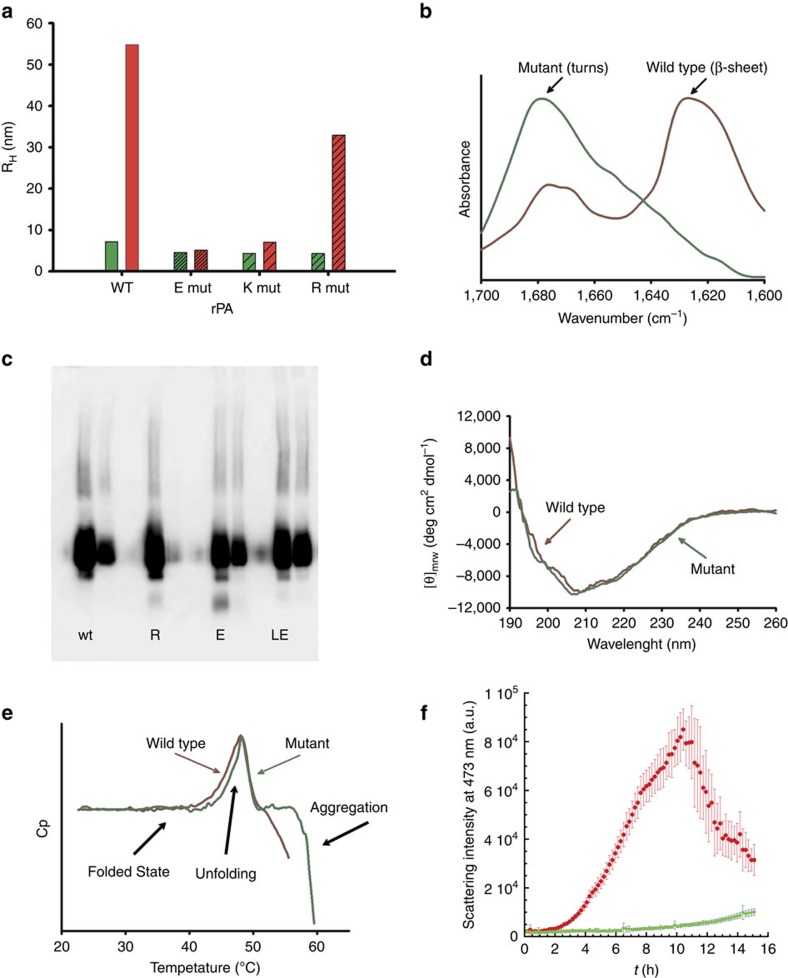
Comparison of the aggregation behaviour of WT and mutant PA. (**a**) The hydrodynamic radius (R_H_) of WT and single Gk mutant PA before (green bar) and after exposure (red bar) to thermal stress at 45 °C for 30 min. (**b**) Infrared amide I band of the WT (NATNIYTVLDKIK) and mutant sequence (NATNIY**E**VLDKIK) surrounding the mutation site of PA APR 1. (**c**) Western blot of WT, single and double mutant PA before and after exposing to thermal stress at 45 °C for 30 min. The lanes 1 and 2 of each sample represent the soluble fraction of PA under normal conditions (25 °C) and after thermal heat stress (40 °C for 30 min). (**d**) Far UV circular dichroism (CD) spectra of the WT and double-mutant (T576E/S559L) PA at 25 °C. (**e**) DSC thermogram of WT (red) and double-mutant (T576E/S559L) PA (green). (**f**) Static light scattering (SLS) at 473 nm measured over time for WT (red) and double-mutant (T576E/S559L) (green) at 40 °C.

Protective antigen (PA) is an element of the protein toxin secreted by *B. anthracis*^28^. It is a key component of recombinant Anthrax vaccines, since the immune response against this virulence factor confers protection against *B. anthracis* infection^29^. However, recombinantly PA (rPA) stored in aqueous solution is aggregation-prone^30^,^31^. The structure of PA reveals four domains^32^. Domain 1 (Fig. 6a, grey) contains two calcium-binding sites and the furin cleavage site required for proteolytic activation of the molecule. Domain 2 (Fig. 6a, red) is required for membrane insertion and together with domain 3 (Fig. 6a, yellow) for heptamer formation. Domain 4 (Fig. 6a, purple) is essential for binding cell surface receptors^28^,^32^. TANGO detects several APRs in the amino acid sequence of the protein (Fig. 6b). It was experimentally demonstrated that the solvent exposed APR (APR1, Fig. 6a,b) in domain 3 is the key determinant of rPA aggregation *in vitro*^33^. Therefore, even though analysis suggests improvements are possible for all three APRs, we focused on APR1 (Fig. 6a,b, N_573_IYTVLD_579_). The MASS plot identified position Thr576 as a suitable design hot spot, allowing for mutation to Glu, Lys and Arg. WT and three mutant proteins were recombinantly expressed in *E. coli* and purified (see methods). The hydrodynamic radius of the proteins was measured using dynamic light scattering (DLS) before and after heat stress (Fig. 7a), revealing a clear increase in the size of WT, consistent with aggregation. Mutants showed a mild (T576R) to strong (T576E and T576K) resistance to size increase upon heat stress suggesting lower aggregation. To corroborate this observation we analysed the T576E mutation in more detail by synthesizing the corresponding WT and mutant APR1 (NATNIYTVLDKIK and NATNIY**E**VLDKIK) as peptides. Analysis of secondary structure of these peptides by Fourier transform infrared spectroscopy (FTIR) (Fig. 7b) reveals the WT peptide displays an amide I band near 1,620 cm^−1^, consistent with aggregation, while the mutant peptide displays a markedly different spectrum with a peak near 1,680 cm^−1^ consistent with a β-turn structure. An additional *in silico* mutation screen using FoldX revealed the stability of the T576E mutant could be improved by 1.2 kcal mol^−1^ by a compensatory mutation S559L. The corresponding double mutant protein was purified and subjected to heat stress followed by native polyacrylamide gel electrophoresis (PAGE) (Fig. 7c), revealing a clearly increased heat resistance compared with the WT and a clear improvement over the single mutation. Far and near UV circular dichroism spectroscopy (CD) (Fig. 7d) and near UV confirmed conservation of the structure between T576E/S559L and WT. Differential Scanning Calorimetry (Fig. 7e) of WT and T576E/S559L revealed only a slight increase in melting temperature Tm of the T576E/S559L mutant (47.8±0.13 °C) compared with the WT (46.4±0.13 °C) by 1.4 °C (*t*-test *P*=0.002), but the narrower transition for the mutant is consistent with a more cooperative unfolding transition. Moveover, whereas WT shows characteristic signals of aggregation in the plot from 50 °C upwards, the T576E/S559L mutant starts aggregating only at a higher temperature, suggesting we have removed the APR responsible for aggregation during storage at room temperature. To further corroborate this observation, we performed a temperature ramped assay, in which we simultaneously measured changes in intrinsic tryptophan fluorescence (ITF) and right-angle light scattering (RALS)^34^,^35^, allowing the combined monitoring of unfolding (Tm, Supplementary Fig. 5A) and aggregation (Tagg, Supplementary Fig. 5B) temperatures in function of a temperature gradient of 0.3 °C min^−1^. The Tm values obtained by this method are similar to those observed by DSC (46.76±0.24 °C and 47.23±0.09 °C for WT and T576E/S559L mutant, respectively). The aggregation onset temperatures differ by 2.4 °C from 47.42±0.01 °C for WT to 49.86±0.01 °C for the T576E/S559L mutant, again in good agreement with the DSC data. Plotting aggregation versus degree of unfolding, it appears the aggregation onset for WT occurs at 53% of the unfolding transition, whereas for the mutant aggregation only initiates at 84% of the unfolding transition (Supplementary Fig. 5C). This is consistent with our design, in which we reduced the aggregation propensity of the strongest and most exposed APR by the T576E mutation, delaying aggregation until more advanced denaturation when the remaining APRs eventually become solvent exposed and initiate aggregation. We also performed similar combined ITF and RALS measurements under isothermal conditions at 40 °C, where both WT and mutant are still on the native baseline in the temperature ramp experiment but close to the unfolding transition. During the first 2 h of this experiment, both mutant and wild type equilibrated to a partially denatured conformation at 38% of the unfolding transtion (Supplementary Fig. 5D). As expected for an aggregation reaction, we observed a lag phase during which the light scattering remains near the baseline, followed by a rapid increase of scattering intensity (Fig. 7f and Supplementary Fig. 5E). During this experiment the aggregation of WT initiates between ∼2 h, whereas the T576E/S559L mutant shows only marginal aggregation over 10 h, demonstrating an increased stress resistance.

**Figure 8:**
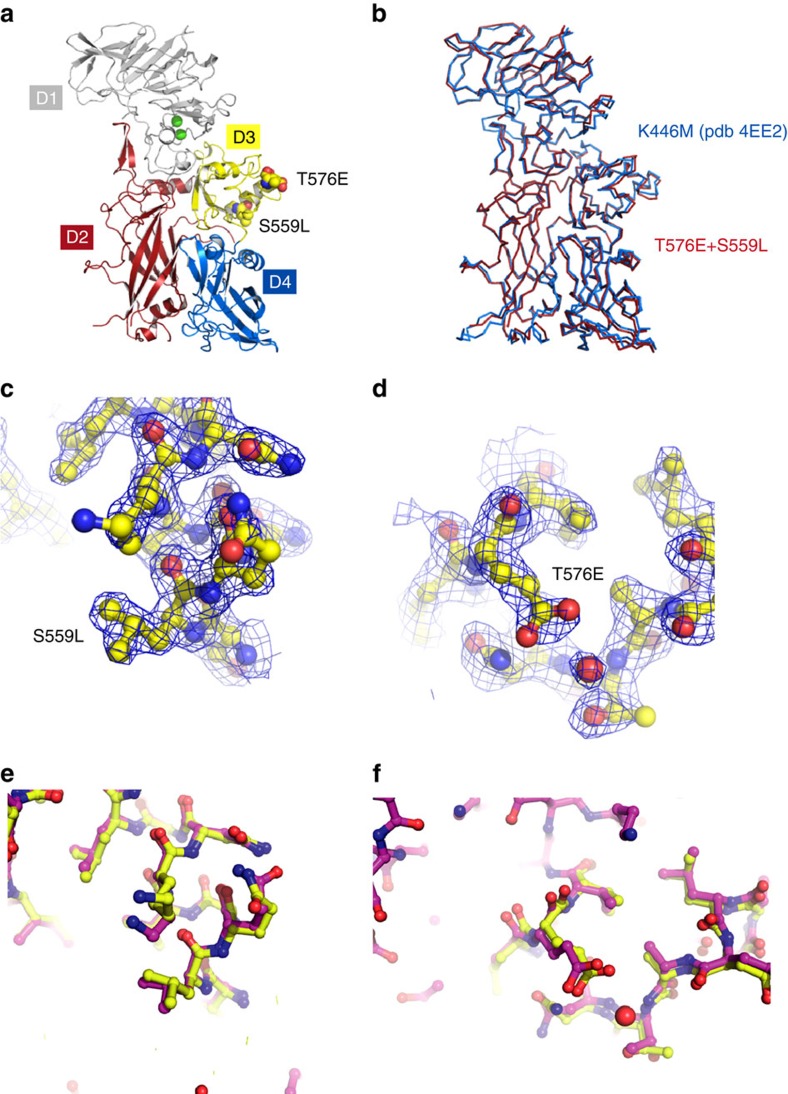
Comparison of the structure of WT and mutant PA. (**a**) Cartoon presentation of APA T576E/S559L. D1, grey is calcium-binding domain. D2, red is membrane insertion and heptamerization domain. D3, yellow is heptamerization domain. D4, blue is receptor binding domain. The positions of the mutated residues are shown in sphere representation. The two green spheres represent calcium ions. (**b**) Superposition of APA T576E/S559L with the published structure of K446M (pdb code 4EE2). The rmsd is 0.78 Å for 675 aligned residues. (**c**,**d**) Experimental electron density maps around S559L and T576E. The blue mesh is a 2*F*_o_*F*_c_ map contoured at a sigma level of 1. The protein is shown in ball and stick representation. (**e**,**f**) Superposition of the crystal structure (yellow) and predicted structure by FoldX (magenta).

To further investigate the effect of the mutations on the protein structure, we solved the crystal structure of the mutant by molecular replacement (1acc (ref. 32)—Fig. 8a) to a resolution of 1.9 Å (Supplementary Table 1) leading to a model that had 0.78 Å rmsd for 675 superposed atoms (Fig. 8b), indicating our mutations were highly conservative of the structure. The mutant side chains were well defined in the electron density (Fig. 8c,d and Supplementary Fig. 6) and superposed well with the predicted side-chain conformations by FoldX (Fig. 8e,f), further validating our approach.

**Figure 9:**
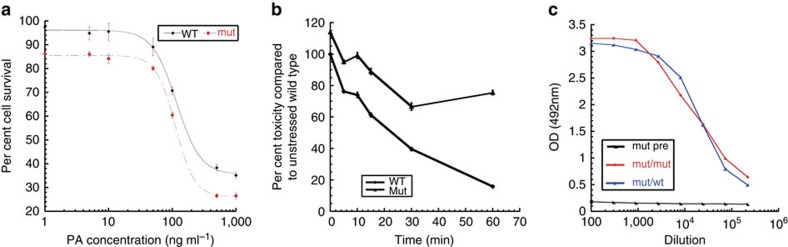
Comparison of the biological activity of WT and mutant PA. (**a**) Concentration dependence of the toxicity of PA in the presence of lethal factor to mouse macrophage Raw264.7 cells. (**b**) Per cent toxicity of PA in the presence of lethal factor to Raw264.7 cells after increasing times of heat stress at 45 °C to the protein sample before the assay. (**c**) Binding of antiserum raised against mutant PA to WT and mutant PA.

We verified the biological function of the protein as well as its ability to raise a protective immune response upon injection in mouse. To assay protein function, we treated Raw264.7 transformed mouse macrophage cells with T576E/S559L mutant and WT rPA in combination with *B. anthracis* protein lethal factor (LF) and determined the lethal dose required to kill 50% of the cells (LD_50_, Fig. 9a). The LD50 values for mutant and WT were identical within error: 116.3±6.5 for WT and 111.6±3.4 for the mutant. However, repeating the assay after increasing times of heat stress at 45 °C, WT activity decreased further than the mutant (Fig. 9b), leading to 20% residual activity of WT after 1 h at 45 °C compared with 80% for the mutant. Finally, we carried out four repeat injection of mutant rPA in mouse (days 0, 7, 10 and 18) and collected blood on day 28 to determine the response titre by ELISA (Fig. 9c). The data show that the naive animals have no anti-rPA antibodies and that after the immunization there is a clear immune response to the mutant and that the mutant antiserum recognizes WT and mutant rPA to comparable level, showing the functionality of mutant rPA as a vaccine component is also conserved. Taken together, our data indicate that mutant T576E/S559L displays a lower aggregation propensity than the WT protein, while conservating both structure and biological activity of the PA protein.

## Discussion

Protein aggregation is one of the main limiting factors for the use of proteins at concentrations far above those for which evolutionary selection has shaped them. Indeed, the global correlation between cellular abundance and protein solubility strongly suggests proteins are operating near their solubility limit^7^. It follows that use of a protein at higher-than-native concentrations requires adaptation of its primary sequence, but it is not immediately apparent how much scope for improvement natural sequences harbour and how many mutations are required for notable improvements. Moreover, the fact that protein abundances are more conserved than mRNA levels could point towards a solubility deadlock for many protein sequences^36^, potentially restricting the scope for artificial protein improvement.

On the other hand, we now understand the basic mechanisms of aggregation. Recently, several reports were made of mutationally reducing protein aggregation through either rational design or directed evolution^37^,^38^,^39^,^40^,^41^,^42^,^43^. The most successful of these mutations involve the introduction of charged residues, which could be due to the increase of the colloidal stability by augmenting the net charge of the protein. On the other hand, these residues are also reminiscent of aggregation gatekeepers. This class of residues was discovered some time ago to flank nearly all naturally occurring APRs^44^. These gatekeepers are usually charged amino acids and Pro, although some reports also include His and Gly^45^. Gatekeepers kinetically control aggregation sufficiently to favour folding. Their placement at the flanks of APRs results from the fact that APRs are usually part of an element of secondary structure in the hydrophobic core of the protein, where charged or structure breaking residues cannot be accommodated. Gatekeepers are therefore enriched at the first possible polypeptide position emerging from the hydrophobic core of the protein.

Our current analysis suggests these earlier analyses may have overlooked one of the most effective forms of aggregation gatekeepers, namely those where placement in the centre of an APR is possible, resulting in its complete suppression, thus rendering it invisible to prediction algorithms. We termed these residues suppressing gatekeepers and a survey of natural polypeptide sequences identified about 1 of these sGKs in every 100 amino acids on average. We find that sGK are more frequently mutated in disease-associated mutations than in polymorphisms, suggesting they have strong impact on protein solubility. However, protein folding and structure put many restrictions on the mutations that can be introduced in a polypeptide, and functional sites provide yet another level of complexity. We were therefore intrigued to find that at least *in silico* the potential for introducing additional suppressing gatekeeper residues is not exhausted in natural sequences, potentially providing a rational way to engineer more soluble protein sequences through reduced aggregation. The crux is finding the few sites where sGKs can be introduced without violating structure. We followed this up for two examples: human α-galactosidase and protective antigen from *B. anthracis* and found that we could reduce aggregation by exploring a very small number of mutations. In both cases, we found back charged amino acids, making it difficult to distinguish effects resulting from increased colloidal stability from those resulting from the elimination of an aggregation prone region. Given that the importance of the latter has been demonstrated in a range of experiments, the effect of the artificial introduction of suppressing gatekeepers is probably an integration of both effects in a single mutation.

Since for the two examples analysed here we managed to introduce residues reducing protein aggregation while conserving both protein structure and function one might wonder why these solutions have not been explored naturally. Of course, the concentration at which these proteins naturally occur may be below their critical concentration for aggregation or they may benefit from chaperone interactions to such an extent that aggregation is not a feature on which selection has to act. Still, it is interesting to note that of all the mutations in α-Gal and rPA that we analysed here, only one (A368P) is a naturally accessible variation, considering single codon changes of the nucleotide sequence. This would imply that even if these proteins would be under selective pressure to increase their solubility, at least this particular set of solutions is not immediately accessible.

## Methods

### Plasmid construction and mutagenesis

The full-length cDNA sequence encoding human α-Gal A (NM_000169) was cloned into the pcDNA4/TO/myc-His vector (Invitrogen). The insert was amplified using primers specific for the human α-Gal gene on Gene Pool cDNA template from human normal skeletal muscle (Invitrogen) with Phusion polymerase (Finnzymes). Then, the PCR product was digested with restriction enzymes Hind III and Xho I and cloned in pcDNA4/TO/myc-His vector to generate an open reading frame encoding α-Gal with a C-terminal Myc-tag. Expression vectors containing single, double and triple mutated α-Gal (D165V, A288D, A346P, A368P, A368R and S405L) were generated by site-directed mutagenesis using sequence-specific primers and PWO DNA polymerase (Roche).

The gene sequence coding for anthrax protective antigen was synthesized by GenScript and subcloned into the bacterial expression vector pET22b+. This vector confers ampicillin resistance and includes the pelB sequence for periplasmic expression. No affinity tag sequence was included. Mutants were made in house using site-directed mutagenesis and mutation was confirmed by DNA sequencing.

### Cell culture and transient transfection

Human cervical cancer cell line HeLa and human osteosarcoma cell line U2OS (used for maximum 20 passages) were cultured in DMEM/F12 medium (Gibco) supplemented with 10% FCS and 1% antibiotics (penicillin/streptomycin) at 37 °C in 5% CO2. For transient transfection in six-well culture plates, 350.000 HeLa cells were plated per well in the medium without antibiotics. An amount 1 μg of plasmid DNA was transfected into HeLa cells using FuGENE HD transfection reagent (Roche) according to manufacturer's protocol. For transient transfection in 96-well culture plates, 6.000 HeLa and U2OS cells were plated per well in the medium without antibiotics. An amount 0.1 μg of plasmid DNA was transfected into the cells using FuGENE HD transfection reagent (Roche) according to manufacturer's protocol. Forty-eight hours after transfection, cells were removed from the incubator and examined.

### SDS–PAGE and western Blot

Forty-eight hours after transfection HeLa cells were lysed in RIPA buffer (1% octylphenoxypolyethoxyethanol (IGEPAL), 0.5% sodium deoxycholate and 0.1% SDS) (Pierce) supplemented with protease inhibitors (Roche) and fractionated by SDS–PAGE (NuPAGE system, Invitrogen). For Western blot the scraped cells were heated with 2% SDS buffer at 99 °C for 10 min, separated using a 10% Bis-Tris gel in MES running buffer and subsequently transferred by electroblotting (fixed current 0.4 A) on a nitrocellulose membrane (Millipore). The membrane was incubated in 5% dried non-fat milk powder dissolved in 0.2% Tris Buffer Saline-Tween (TBST) for 1 h at room temperature and subsequently incubated with primary mouse anti-myc antibody (Invitrogen) followed by incubation by secondary goat HRP-conjugated anti-mouse IgG (Promega). Proteins were visualized using chemiluminescence immunoblotting detection reagent (ECL, Millipore).

### Size exclusion chromatography

For the analysis of the α-Gal aggregation state using SEC, transfected HeLa cells were lysed in RIPA buffer supplemented with protease inhibitors, centrifuged 5 min at 3,000 r.p.m. and 400 μl of the supernatant was subsequently loaded onto a Superdex S200 HR10/30 column (GE Healthcare) equilibrated in hypotonic buffer (20 mM HEPES, 10 mM KCl, 1 mM MgCl2, 1 mM EDTA, 1 mM EGTA, 1 mM DTT and pH 7.5). Eluted fractions were concentrated by 20% trichloroacetic acid precipitation, washed with acetone and analysed by SDS–PAGE. The bands densities were quantified using the Quantity One program from the ChemiDoc System (Bio-Rad). A mixture of molecular weight markers (Bio-Rad) was injected onto the column as a gel filtration standard.

### Enzymatic assay

The activity of α-Gal was determined by fluorogenic substrate 4-methylumbelliferyl-α-D-galactopyranoside (5 mM 4-MU-α-Gal) as described previously^46^. *N*-acetylgalactosamine (D-GalNAc) was used as an inhibitor of α-Gal B activity. α-Gal B is a second α-Gal in the cells that hydrolyses the artificial substrate but its activity in FD patients is normal or increased. In brief, HeLa cells transfected with WT or mutant α-Gal were harvested and lysed in PBS by three cycles of freezing/thawing in acetone-dry ice water bath. The supernatant obtained by centrifugation at 10,000*g* was incubated with substrate solution (5 mM 4-MU-α-Gal and 100 mM D-GalNAc in 0.1 M citrate buffer pH 4.5) at 37 °C and the fluorescence was measured in a plate reader (POLARstar OPTIMA, BMG Labtech) within an hour. The slope of the linear part of the substrate conversion curve was a measure of the concentration of active enzyme in the lysates. α-Gal concentration in the whole-cell lysates was determined by Western blot. To determine the enzymatic activity, the assays were performed in three independent experiments.

### Protein crystallization and X-ray structure determination

APA S559L/T576E protein solution was concentrated to 9.6 mg ml^−1^ and crystallization screens were set up using a Mosquito nanoliter crystallization robot (TTP Labtech). Plate-shaped crystals appeared within a couple of days and grew at 4 °C to >100 μm in size within one week under the following conditions: 20 mM MgCl_2_; 100 mM HEPES pH 7.5; and 22% poly(acrylic acid sodium salt) 5,100. Crystals were flash-cooled in liquid nitrogen after cryo-protection with 30% glycerol.

X-ray diffraction data were collected to a resolution of 1.94 Å at the X06A beam line of the Swiss Light Source (Paul Scherrer Institute, Villigen, Switzerland). Full crystallographic and refinement statistics are reported in Supplementary Table 1. Diffraction data were processed with XDS^47^ and scaled with SCALA in the CCP4 suite^48^. The structure was solved using molecular replacement in PHASER using the published APA structure^32^ (pdb accession code 1ACC) as a search model. The obtained model was automatically rebuilt using Autobuild in the PHENIX suite^49^. Iterative cycles of manual model building and refinement in COOT^50^ and PHENIX were carried out until the R-factors converged to a value of 17.8% and 22.7% (R_work_ and R_free_, respectively). The higher resolution structure of the published APA K446M structure^51^ (pdb accession code 4EE2) was used to guide rebuilding of surface-exposed loops with poor electron density. The final model includes residues S14-S163, T169-D276, R287-V303, V320-S339 and E343-G735. All omitted regions correspond to surface-exposed loops that have poor electron density. Model validation was done using MOLPROBITY^52^ and all figures were prepared using PyMOL (Schrödinger).

### Statistical analysis

To confirm the consistency of the results, all described experiments were performed in minimum three separate replicates. For statistical evaluation of the determined averages and standard deviations of the mean, data were analysed for significant differences using unpaired Student's *t*-test with a *P* value <0.05 (*P*<0.05). Asterisks indicating the level of the *P* value centred over the error bar mean: ‘*' *P*< 0.05, ‘**' *P*< 0.01, ‘***' *P*< 0.001 and ‘****' *P*<0.0001.

### Bio-informatics analysis

TANGO^13^ was used to determine APRs in human proteins. This resulted in an aggregation propensity (0–100%) for each residue, whereby an aggregating segment is defined as a continuous stretch of at least five consecutive residues, each with a TANGO score higher than 5%. As each APR has an average TANGO score, ranging from 0 to 100, aggregating segments are binned based on this average value. All modelling was performed using the FoldX 3b5.1 force field^23^ and tool suite and structural visualization was achieved using Yasara^53^.

### Immunization

Polyclonal antibodies were raised in rat via a 28 days Speedy Polyclonal Antibody program at Eurogentec. WT and mutant PA were prepared for immunization as follows. Under sterile conditions alhydrogel aluminium hydroxide gel adjuvant (Brenntag) (conc. 10 mg ml^−1^=1%) was washed three times in 9 mM sodium phosphate pH 7.3, 137 mM NaCl, 12 mM KCl and finally resuspended in this buffer at a concentration of 1%. Protein was filtered through a 100 μm filter and the buffer exchanged to 9 mM sodium phosphate pH 7.3, 137 mM NaCl, 12 mM KCl (also sterile filtered). Protein concentration was calculated using a theoretical extinction coefficient of 80,200. Protein and adjuvant were mixed by rotating at 4 °C for one hour to a final concentration of 2.6 mg ml^−1^ (0.26%) adjuvant and 0.2 mg ml^−1^ PA protein, transferred to sterile glass vials and sent to Eurogentec. Upon completion of the 28 days Speedy program Eurogentec performed antiserum analysis by an indirect ELISA assay against WT PA to compare pre-immune serum with immunized serum.

### Protein expression

An amount 20 μl calcium competent BL21 (DE3) cells were transformed with 10 ng plasmid DNA (WT or mutant), plated on LB-agar plus 100 μg ml^−1^ ampicillin, and incubated overnight at 37 °C. The following day a single colony was used to inoculate 10 ml LB medium plus 100 μg ml^−1^ ampicillin and incubated overnight at 37 °C, shaking. The next day this overnight culture was used to inoculate 1,000 ml of LB medium plus 100 μg ml^−1^ ampicillin. Cells were grown at 37 °C plus shaking until an OD of 0.4–0.6 was reached, at which point the cultures were cooled to 25 °C over a period of 1 h. Protein expression was induced by the addition of 1 mM IPTG and additional ampicillin was added at 100 μg ml^−1^. Protein expression was allowed to proceed overnight, cells were harvested by centrifugation and bacterial cell pellets stored at −80 °C before protein purification.

### Protein purification

Bacterial cell pellets were recovered from −80 °C and thawed on ice. Ice cold lysis buffer was added at a ratio of 50 ml per 1,000 ml of bacterial culture pelleted and the combination gently stirred for 1 h at 4 °C. Lysis buffer; 50 mM Tris-HCl pH 7.8, 1 mM EDTA, 30 mM Benzamidine HCl, plus the necessary amount of protease inhibitor tablets (complete ultra, mini EDTA-free, Roche); pH was adjusted to pH 7.8 and the buffer supplemented to 25% sucrose. 1 mg ml^−1^ lysozyme (from chicken egg white, Fluka) was added and Universal Nuclease (Pierce) were also added at 1:10,000. Cell lysate was centrifuged at 32,800*g* for 20 min (4 °C), and the supernatants collected. An equal volume of ice cold 20 mM Hepes pH 7.0 was added and the samples were kept on ice. Supernatant was subjected to anion exchange chromatography (HiTrap Q HP, 5 ml, GE Healthcare) which was equilibrated with five column volumes 20 mM Hepes pH 7.0. Protein was eluted with a 0–250 mM NaCl gradient, anthrax protective antigen eluted at 80 mM NaCl. Anthrax Protective Antigen was immediately diluted 1:1 with ice-cold 2.5 mM K_2_HPO_4_ and then applied to a hydroxyapatite column (CHT Ceramic Hydroxyapetite, 5 ml, Bio-Rad) equilibrated with 10 column volumes 2.5 mM K_2_HPO_4_. Protein was eluted with a 2.5–250 mM K_2_HPO_4_gradient, anthrax protective antigen eluted at 5 mM K_2_HPO_4_. Both chromatographies were performed at 4 °C. Protein was concentrated by centrifugation using a molecular weight cutoff of 30,000 Da, simultaneously exchanging the buffer to 1 × PBS, 0.02% tween, 500 mM sucrose. Protein was flash frozen in liquid nitrogen and stored at −80 °C

### Lethal factor toxicity assay

Raw 264.7 cells were seeded at 30 K cells/well in a 96-well plate and the following day a toxicity assay was performed as follows. WT and mutant PA were diluted to 1 mg ml^−1^ in 1 × PBS and samples were heated for 0, 5, 15, 30 and 60 min in a 45 °C heat block. Samples were incubated on ice and immediately filtered through SpinX cellulose acetate filters (Sigma). An equal volume was taken from all samples, based on the start concentration of 1 mg ml^−1^, to achieve a dilution of 500 ng  ml^−1^ in DMEM (plus 10% FCS and antibiotics) containing 2,000 ng ml^−1^ Anthrax Lethal Factor, Recombinant (Calbiochem #176900) (LF). Medium was aspirated from the cells and protein/lethal factor combinations were added at 100 μl per well. Controls for this assay were: (a) zero PA zero LF; and (b) zero PA, 2,000 ng  ml^−1^ LF, 50 μg  ml^−1^ Mellitin (Sigma, M2272-1MG), medium alone (no cells). After an incubation period of 4 h at 37 °C toxicity was assayed using the CellTiter 96 Aqueous One Solution Cell Proliferation Assay (G3580, Promega) as per the manufactures instructions. In addition, the supernatants of the heated samples were probed by Western blot. An amount of 2 μl of protein sample was subjected to SDS–PAGE using Bio-Rad any kD TGX gels and transferred to nitrocellulose membrane using a Bio-Rad Turbo Blot system. The membrane was blocked overnight with 1 × PBS, 1% BSA, 0.05%Tween. Primary antibody (mouse mAb to PA (BAP0101) Abcam ab1988) was diluted 1/2,000 diluted in blocking buffer and incubated for 1 h. The membrane was washed 3 × 10 min with 1 × PBS, 0.05% Tween. Secondary antibody (anti mouse HRP: W4021, Promega) was diluted 1/2,000 diluted in blocking buffer and incubated for 1 h. The membrane was washed 3 × 10 min with PBS 0.05% Tween and 1 × 10 min with ultra pure water. Protein was visualized using Immobilon Western Chemiluminescent HRP substrate (WBKLS0050, Millipore).

### Biophysical characterization

DLS measurements were made at room temperature with a DynaPro DLS plate reader instrument (Wyatt, Santa Barbara, CA, USA) equipped with a 830-nm laser source. Samples were placed into a flat-bottom 96-well microclear plate (Greiner, Frickenhausen, Germany). The autocorrelation of scattered light intensity at a 90° angle was recorded for 10 s and averaged over 40 recordings to obtain a single data point. The Wyatt Dynamics software was used to calculate the hydrodynamic radius by assuming a spherical particle shape. Attenuated total reflection FTIR was performed using a Bruker Tensor 27 infrared spectrophotometer equipped with a Bio-ATR II accessory. Spectra were recorded in the range of 900–3,500 cm^−1^ at a spectral resolution of 4 cm^−1^ by accumulating 120 data acquisitions. The spectrophotometer was continuously purged with dried air. Spectra were corrected for atmospheric interference, baseline-subtracted, and rescaled in the amide II area (1,500 to 1,600 cm^−1^). Circular dichroism (CD) spectra were recorded using a Jasco J-715 spectropolarimeter (Jasco, Easton, MD, USA) equipped with a temperature-controlled cell holder thermostated at 25 °C. The far UV and near UV CD spectra of PA in PBS were recorded in a cylindrical quartz cuvette (Hellma) of path length 0.01 and 0.5 cm, respectively. Each CD spectra is an average of 5 scans recorded at a scan speed of 50 nm min^−1^ (far UV) and 20 nm min^−1^ (near UV). The data was baseline corrected for buffer absorption, smoothed using Savitzky-Golay algorithm and converted to mean residual weight units. Tm and Tagg of were determined simultaneously using ITF and RALS using the OPTIM 1000 (Unchained Labs, Boston, USA) instrument. All experiments were performed in triplicates of triplicates. For the temperature ramped experiments a linear ramp was done with a increase of 0.3 °C min^−1^. For the isothermal experiments a temperature of 40 °C was maintained. Exposure time was set to 1,000 ms, slit width was 100 μm. The barycentric mean and scattering at 266 and 475 nm were calculated and plotted using R-studio.

### DSC measurements

The thermal unfolding transition mid-point temperature (*Tm*) of WT and mutant rPA was measured using a Micro-Cal VP DSC. A 0.516 ml solution of WT or mutant rPA (0.5 mg ml^−1^) dissolved in 10 mM PBS pH 7.4 was loaded in the Micro-Cal sample cell and thermograms were recorded at a scan rate of 1 °C min^−1^ with PBS in the reference cell. Thermograms were analysed and curve fitted using Micro-Cal Origin 7.0 software. The experiments were performed in triplicates using freshly prepared rPA samples.

### Agal fractionation

HeLa cells were cultured in DMEM medium supplemented with 10% FBS, 1 mM sodium pyruvate and non-essential amino acids. For transient transfection in 6-well culture plates, 300,000 HeLa cells were plated per well and 1.5 μg of plasmid DNA was transfected into the cells using Lipofectamine 2,000 transfection reagent (Life technologies). Twenty-four hours after transfection, cells were wash with cold PBS and lysed in buffer (1% octylphenoxypolyethoxyethanol (IGEPAL), 0.1 M Dithiothreitol (DTT) and 3 mM NaCl in Tris pH 8) supplemented with protease inhibitors (Roche). Lysates were centrifuged for 15 min at 13.400 r.p.m. at 4 °C and the supernatant was separated from the pellet. The pellets were washed with lysis buffer and dissolved in 8 M urea for 1 h. For Western blot, samples were boiled in sample buffer (2% SDS and 1% 2-Mercaptoethanol) for 10 min, run on Any Kd gels (Biorad) and transferred to nitrocellulose membranes (Biorad). The membranes were blocked for 1 h with 5% milk powder dissolved in 0.2% TBST, incubated with primary mouse anti-myc antibody (Life technologies, 46-0603) or primary mouse anti-GAPDH antibody (Santa Cruz, SC-32233), followed by incubation with secondary goat HRP-conjugated anti-mouse antibody (Promega). Proteins were visualized using chemiluminescence.

## Additional information

**Accession codes**: Coordinates and structure factors for PA mutant T576E/S559L have been deposited in the RCSB Protein Data Bank under accession codes 5fr3.

**How to cite this article:** Ganesan, A. *et al*. Structural hot spots for the solubility of globular proteins. *Nat. Commun.* 7:10816 doi: 10.1038/ncomms10816 (2016).

## Supplementary Material

Supplementary Information Supplementary Figures 1-6, Supplementary Table 1 and Supplementary Reference

## Figures and Tables

**Table 1:** Mutations with predicted beneficial effect on thermodynamic stability >2 kcal ml^−1^ obtained by FoldX analysis.

pos	wt	mut	difTANGO	difstab
405	S	L	−0,03	−3,34
405	S	M	−0,02	−3,09
333	Q	R	1,8	−2,62
368	A	P	−91,8	−2,31
408	N	P	0,1	−2,27
327	Q	M	0,0	−2,20

## References

[b1] Mehdi A. M., Patrick R., Bailey T. L. & Boden M. Predicting the dynamics of protein abundance. Mol. Cell. Proteomics 13, 1330–1340 (2014). 24532840 10.1074/mcp.M113.033076 PMC4014288

[b2] Ellis R. J. Macromolecular crowding: obvious but underappreciated. Trends Biochem. Sci. 26, 597–604 (2001). 11590012 10.1016/s0968-0004(01)01938-7

[b3] Tartaglia G. G. & Vendruscolo M. Correlation between mRNA expression levels and protein aggregation propensities in subcellular localisations. Mol. Biosyst. 5, 1873–1876 (2009). 19763336 10.1039/b913099n

[b4] Tartaglia G. G., Pechmann S., Dobson C. M. & Vendruscolo M. A relationship between mRNA expression levels and protein solubility in *E. coli*. J. Mol. Biol. 388, 381–389 (2009). 19281824 10.1016/j.jmb.2009.03.002

[b5] Castillo V., Grana-Montes R. & Ventura S. The aggregation properties of *Escherichia coli* proteins associated with their cellular abundance. Biotechnol. J. 6, 752–760 (2011). 21538899 10.1002/biot.201100014

[b6] Tartaglia G. G., Pechmann S., Dobson C. M. & Vendruscolo M. Life on the edge: a link between gene expression levels and aggregation rates of human proteins. Trends Biochem. Sci. 32, 204–206 (2007). 17419062 10.1016/j.tibs.2007.03.005

[b7] Tartaglia G. G. & Vendruscolo M. Proteome-level interplay between folding and aggregation propensities of proteins. J. Mol. Biol. 402, 919–928 (2010). 20709078 10.1016/j.jmb.2010.08.013

[b8] De Baets G. . An evolutionary trade-off between protein turnover rate and protein aggregation favors a higher aggregation propensity in fast degrading proteins. PLoS Comput. Biol. 7, e1002090 (2011). 21731483 10.1371/journal.pcbi.1002090 PMC3121684

[b9] Linding R., Schymkowitz J., Rousseau F., Diella F. & Serrano L. A comparative study of the relationship between protein structure and beta-aggregation in globular and intrinsically disordered proteins. J. Mol. Biol. 342, 345–353 (2004). 15313629 10.1016/j.jmb.2004.06.088

[b10] Esteras-Chopo A., Serrano L. & de la Paz M. L. The amyloid stretch hypothesis: recruiting proteins toward the dark side. Proc. Natl Acad. Sci. USA 102, 16672–16677 (2005). 16263932 10.1073/pnas.0505905102 PMC1283810

[b11] Teng P. K. & Eisenberg D. Short protein segments can drive a non-fibrillizing protein into the amyloid state. Protein Eng. Des. Sel. 22, 531–536 (2009). 19602569 10.1093/protein/gzp037 PMC2719503

[b12] Ventura S. . Short amino acid stretches can mediate amyloid formation in globular proteins: the Src homology 3 (SH3) case. Proc. Natl Acad. Sci. USA 101, 7258–7263 (2004). 15123800 10.1073/pnas.0308249101 PMC409906

[b13] Fernandez-Escamilla A. M., Rousseau F., Schymkowitz J. & Serrano L. Prediction of sequence-dependent and mutational effects on the aggregation of peptides and proteins. Nat. Biotechnol. 22, 1302–1306 (2004). 15361882 10.1038/nbt1012

[b14] Goldschmidt L., Teng P. K., Riek R. & Eisenberg D. Identifying the amylome, proteins capable of forming amyloid-like fibrils. Proc. Natl Acad. Sci. USA 107, 3487–3492 (2010). 20133726 10.1073/pnas.0915166107 PMC2840437

[b15] Agostini F., Vendruscolo M. & Tartaglia G. G. Sequence-based prediction of protein solubility. J. Mol. Biol. 421, 237–241 (2012). 22172487 10.1016/j.jmb.2011.12.005

[b16] Hooft R. W., Sander C., Scharf M. & Vriend G. The PDBFINDER database: a summary of PDB, DSSP and HSSP information with added value. Comput. Appl. Biosci. 12, 525–529 (1996). 9021272 10.1093/bioinformatics/12.6.525

[b17] Niwa T. . Bimodal protein solubility distribution revealed by an aggregation analysis of the entire ensemble of *Escherichia coli* proteins. Proc. Natl Acad. Sci. USA 106, 4201–4206 (2009). 19251648 10.1073/pnas.0811922106 PMC2657415

[b18] Vogel C. & Marcotte E. M. Insights into the regulation of protein abundance from proteomic and transcriptomic analyses. Nat. Rev. Genet. 13, 227–232 (2012). 22411467 10.1038/nrg3185 PMC3654667

[b19] Rousseau F., Serrano L. & Schymkowitz J. W. How evolutionary pressure against protein aggregation shaped chaperone specificity. J. Mol. Biol. 355, 1037–1047 (2006). 16359707 10.1016/j.jmb.2005.11.035

[b20] Rousseau F. & Serrano L. Schymkowitz JWH. How evolutionary pressure against protein aggregation shaped chaperone specificity. J. Mol. Biol. 355, 1037–1047 (2006). 16359707 10.1016/j.jmb.2005.11.035

[b21] De Baets G., Van Durme J., Rousseau F. & Schymkowitz J. A genome-wide sequence-structure analysis suggests aggregation gatekeepers constitute an evolutionary constrained functional class. J. Mol. Biol. 426, 2405–2412 (2014). 24735868 10.1016/j.jmb.2014.04.007

[b22] Hooft R. W. W., Sander C. & Vriend G. Verification of protein structures: side-chain planarity. J. Appl. Crystallogr. 29, 714–716 (1996).

[b23] Schymkowitz J. . The FoldX web server: an online force field. Nucleic Acids Res. 33, W382–W388 (2005). 15980494 10.1093/nar/gki387 PMC1160148

[b24] Wang M. . PaxDb, a database of protein abundance averages across all three domains of life. Mol. Cell. Proteomics 11, 492–500 (2012). 22535208 10.1074/mcp.O111.014704 PMC3412977

[b25] Brady R. O. . Enzymatic defect in Fabry's disease. Ceramidetrihexosidase deficiency. N. Engl. J. Med. 276, 1163–1167 (1967). 6023233 10.1056/NEJM196705252762101

[b26] Eng C. M. & Desnick R. J. Molecular basis of Fabry disease: mutations and polymorphisms in the human alpha-galactosidase A gene. Hum. Mutat. 3, 103–111 (1994). 7911050 10.1002/humu.1380030204

[b27] Siekierska A. . Alpha-galactosidase aggregation is a determinant of pharmacological chaperone efficacy on Fabry disease mutants. J. Biol. Chem. 287, 28386–28397 (2012). 22773828 10.1074/jbc.M112.351056 PMC3436532

[b28] Collier R. J. Membrane translocation by anthrax toxin. Mol. Aspects Med. 30, 413–422 (2009). 19563824 10.1016/j.mam.2009.06.003 PMC2783560

[b29] Brey R. N. Molecular basis for improved anthrax vaccines. Adv. Drug. Deliv. Rev. 57, 1266–1292 (2005). 15935874 10.1016/j.addr.2005.01.028

[b30] Singh S. . Thermal inactivation of protective antigen of *Bacillus anthracis* and its prevention by polyol osmolytes. Biochem. Biophys. Res. Commun. 322, 1029–1037 (2004). 15336568 10.1016/j.bbrc.2004.08.020

[b31] Chalton D. A. . Unfolding transitions of *Bacillus anthracis* protective antigen. Arch. Biochem. Biophys. 465, 1–10 (2007). 17531947 10.1016/j.abb.2007.04.030

[b32] Petosa C., Collier R. J., Klimpel K. R., Leppla S. H. & Liddington R. C. Crystal structure of the anthrax toxin protective antigen. Nature 385, 833–838 (1997). 9039918 10.1038/385833a0

[b33] Ganesan A., Watkinson A. & Moore B. D. Biophysical characterisation of thermal-induced precipitates of recombinant anthrax protective antigen: evidence for kinetically trapped unfolding domains in solid-state. Eur. J. Pharm. Biopharm. 82, 475–484 (2012). 22683695 10.1016/j.ejpb.2012.05.019

[b34] Garidel P., Hegyi M., Bassarab S. & Weichel M. A rapid, sensitive and economical assessment of monoclonal antibody conformational stability by intrinsic tryptophan fluorescence spectroscopy. Biotechnol. J. 3, 1201–1211 (2008). 18702089 10.1002/biot.200800091

[b35] Karow A. R., Gotzl J. & Garidel P. Resolving power of dynamic light scattering for protein and polystyrene nanoparticles. Pharm. Dev. Technol. 20, 84–89 (2015). 24773236 10.3109/10837450.2014.910808

[b36] Laurent J. M. . Protein abundances are more conserved than mRNA abundances across diverse taxa. Proteomics 10, 4209–4212 (2010). 21089048 10.1002/pmic.201000327 PMC3113407

[b37] Sahin E. . Computational design and biophysical characterization of aggregation-resistant point mutations for gamma D crystallin illustrate a balance of conformational stability and intrinsic aggregation propensity. Biochemistry 50, 628–639 (2011). 21184609 10.1021/bi100978r

[b38] Zhang A., Singh S. K., Shirts M. R., Kumar S. & Fernandez E. J. distinct aggregation mechanisms of monoclonal antibody under thermal and freeze-thaw stresses revealed by hydrogen exchange. Pharm. Res. 29, 236–250 (2012). 21805212 10.1007/s11095-011-0538-y

[b39] Buck P. M. . Computational methods to predict therapeutic protein aggregation. Methods. Mol. Biol. 899, 425–451 (2012). 22735968 10.1007/978-1-61779-921-1_26

[b40] Perchiacca J. M., Bhattacharya M. & Tessier P. M. Mutational analysis of domain antibodies reveals aggregation hotspots within and near the complementarity determining regions. Proteins 79, 2637–2647 (2011). 21732420 10.1002/prot.23085

[b41] Jespers L., Schon O., Famm K. & Winter G. Aggregation-resistant domain antibodies selected on phage by heat denaturation. Nat. Biotechnol. 22, 1161–1165 (2004). 15300256 10.1038/nbt1000

[b42] Perchiacca J. M., Lee C. C. & Tessier P. M. Optimal charged mutations in the complementarity-determining regions that prevent domain antibody aggregation are dependent on the antibody scaffold. Protein Eng. Des. Sel. 27, 29–39 (2014). 24398633 10.1093/protein/gzt058

[b43] Rouet R., Lowe D. & Christ D. Stability engineering of the human antibody repertoire. FEBS Lett. 588, 269–277 (2014). 24291820 10.1016/j.febslet.2013.11.029

[b44] De Baets G., Van Durme J., Rousseau F. & Schymkowitz J. A genome-wide sequence-structure analysis suggests aggregation gatekeepers constitute an evolutionary constrained functional class. J. Mol. Biol. 526, 2405–2412 (2014). 24735868 10.1016/j.jmb.2014.04.007

[b45] Monsellier E. & Chiti F. Prevention of amyloid-like aggregation as a driving force of protein evolution. EMBO. Rep. 8, 737–742 (2007). 17668004 10.1038/sj.embor.7401034 PMC1978086

[b46] Mayes J. S., Scheerer J. B., Sifers R. N. & Donaldson M. L. Differential assay for lysosomal alpha-galactosidases in human tissues and its application to Fabry's disease. Clin. Chim. Acta 112, 247–251 (1981). 6263521 10.1016/0009-8981(81)90384-3

[b47] Kabsch W. XDS. Acta Crystallogr. D. Biol. Crystallogr. 66, 125–132 (2010). 20124692 10.1107/S0907444909047337 PMC2815665

[b48] Winn M. D. . Overview of the CCP4 suite and current developments. Acta Crystallogr. D. Biol. Crystallogr. 67, 235–242 (2011). 21460441 10.1107/S0907444910045749 PMC3069738

[b49] Adams P. D. . PHENIX: a comprehensive Python-based system for macromolecular structure solution. Acta. Crystallogr. D. Biol. Crystallogr. 66, 213–221 (2010). 20124702 10.1107/S0907444909052925 PMC2815670

[b50] Emsley P., Lohkamp B., Scott W. G. & Cowtan K. Features and development of Coot. Acta. Crystallogr. D. Biol. Crystallogr. 66, 486–501 (2010). 20383002 10.1107/S0907444910007493 PMC2852313

[b51] Kintzer A. F., Tang I. I., Schawel A. K., Brown M. J. & Krantz B. A. Anthrax toxin protective antigen integrates poly-gamma-D-glutamate and pH signals to sense the optimal environment for channel formation. Proc. Natl Acad. Sci. USA 109, 18378–18383 (2012). 23100533 10.1073/pnas.1208280109 PMC3494962

[b52] Chen V. B. . MolProbity: all-atom structure validation for macromolecular crystallography. Acta. Crystallogr. D. Biol. Crystallogr. 66, 12–21 (2010). 20057044 10.1107/S0907444909042073 PMC2803126

[b53] Krieger E., Koraimann G. & Vriend G. Increasing the precision of comparative models with YASARA NOVA--a self-parameterizing force field. Proteins 47, 393–402 (2002). 11948792 10.1002/prot.10104

[b54] Lieberman R. L., D'Aquino J. A., Ringe D. & Petsko G. A. Effects of pH and iminosugar pharmacological chaperones on lysosomal glycosidase structure and stability. Biochemistry 48, 4816–4827 (2009). 19374450 10.1021/bi9002265 PMC2699628

