# Supplementary material for: Structural hot spots for the solubility of globular proteins
Source: Nat Commun. 2016 Feb 24;7:10816. doi: 10.1038/ncomms10816 (PMC4770091; doi:10.1038/ncomms10816)
Supplement: Supplementary Information — Supplementary Figures 1-6, Supplementary Table 1 and Supplementary Reference [file ncomms10816-s1.pdf]

Supplementary Information

Supplementary Figures

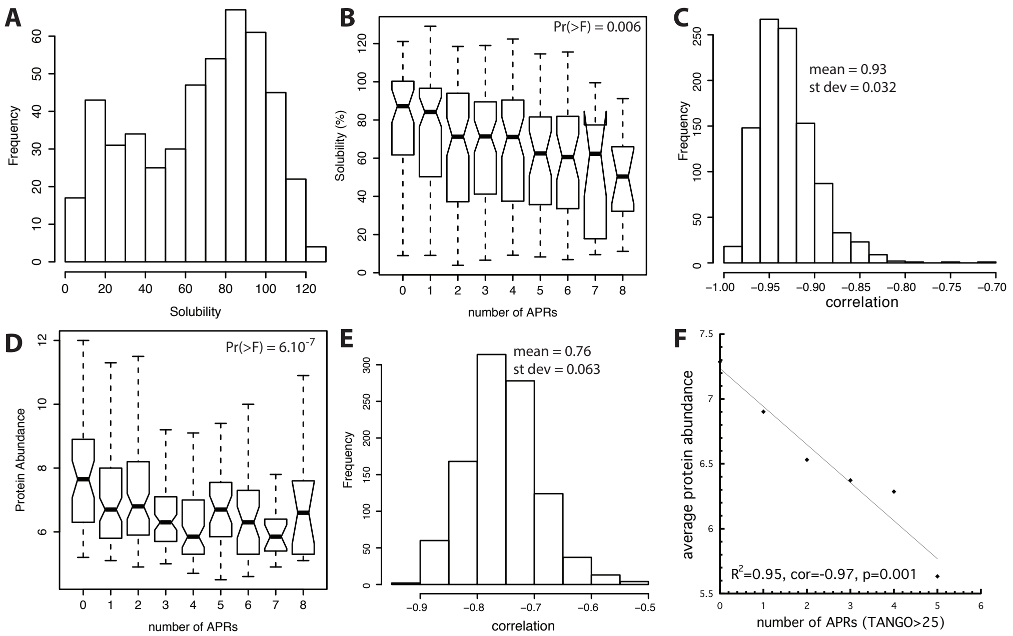

Supplementary Figure 1 – Statistical analysis of Solubility and Abundance correlation. (A) Histogram of the protein solubilities in the dataset shows a bimodal distribution (data from Taguchi and co-workers[1]). **(B)** Boxplots of protein solubility values of proteins categorized by their per number of aggregation prone regions. The p-value is from an ANOVA analysis. **(C)** Histogram of the correlation values obtained from 1000-fold bootstrapping the correlation observed in Figure 1A using random samples consisting of 80% of the data. **(D)** Boxplots of protein abundance values of proteins categorized by their number of aggregation prone regions. The p-value is from an ANOVA analysis. **(E)** Histogram of the correlation values obtained from 1000-fold bootstrapping the correlation observed in Figure 2A using random samples consisting of 80% of the data. **(F)** Similar to Figure 1B, but at higher TANGO cutoff. The scatterplot of the average experimentally determined abundance of *E. coli* proteins grouped by the number of APRs (TANGO >25).

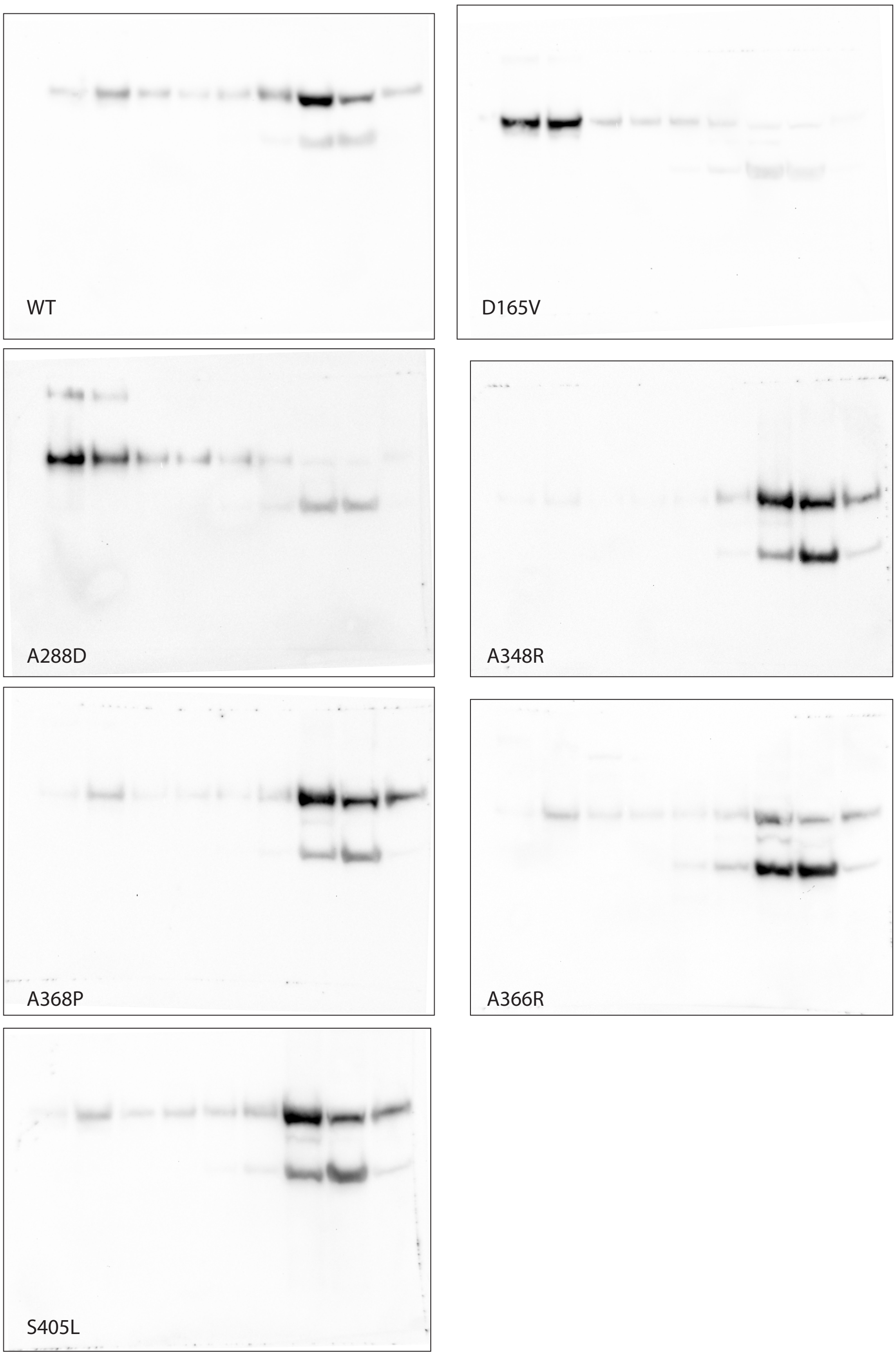

Supplementary Figure 2: Full western blot images of the Size Exclusion Chromatography data in Figure 4A.

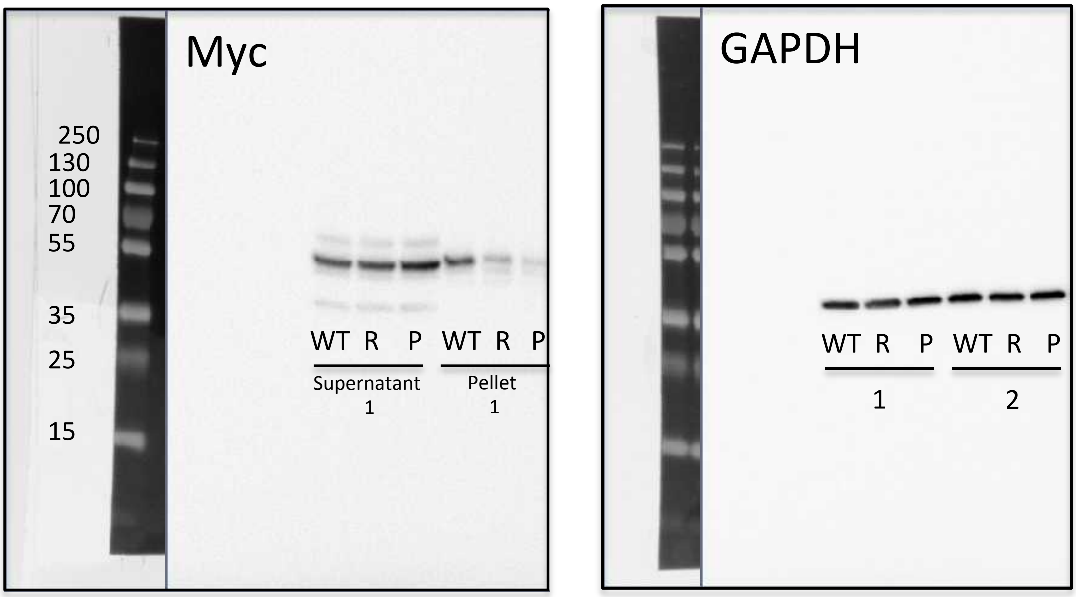

Supplementary Figure 3: Full western blot images of Figure 5B.

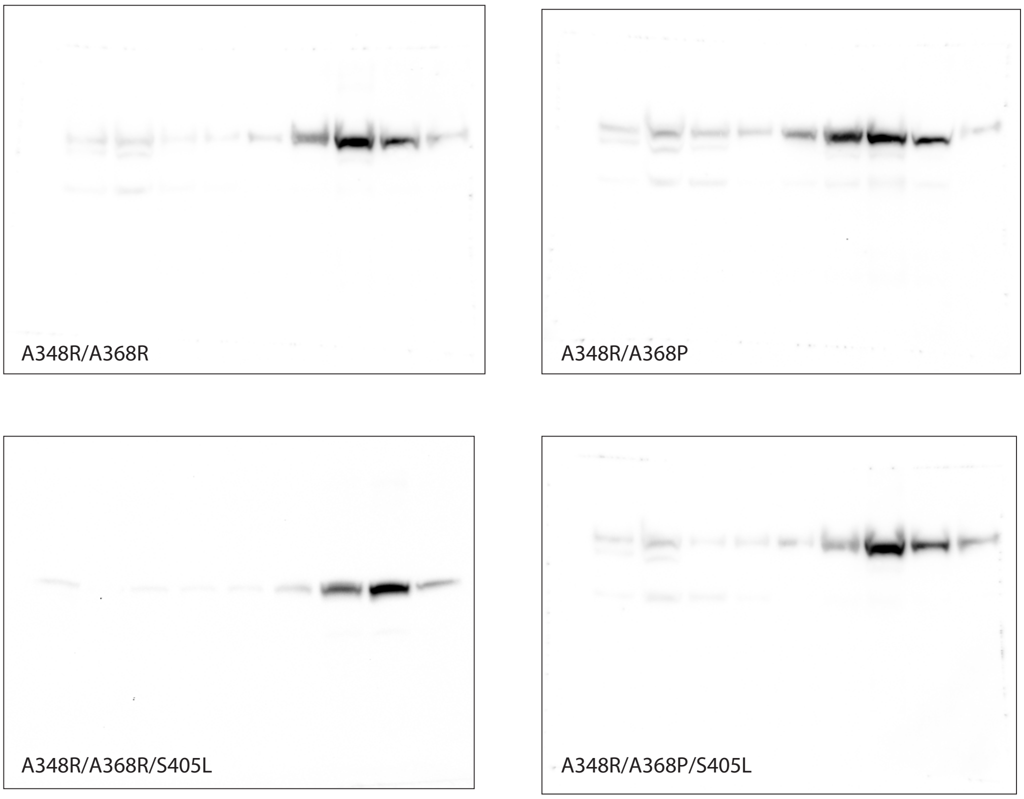

Supplementary Figure 4: Full western blot image of the Size Exclusion Chromatography data of Figure 5C.

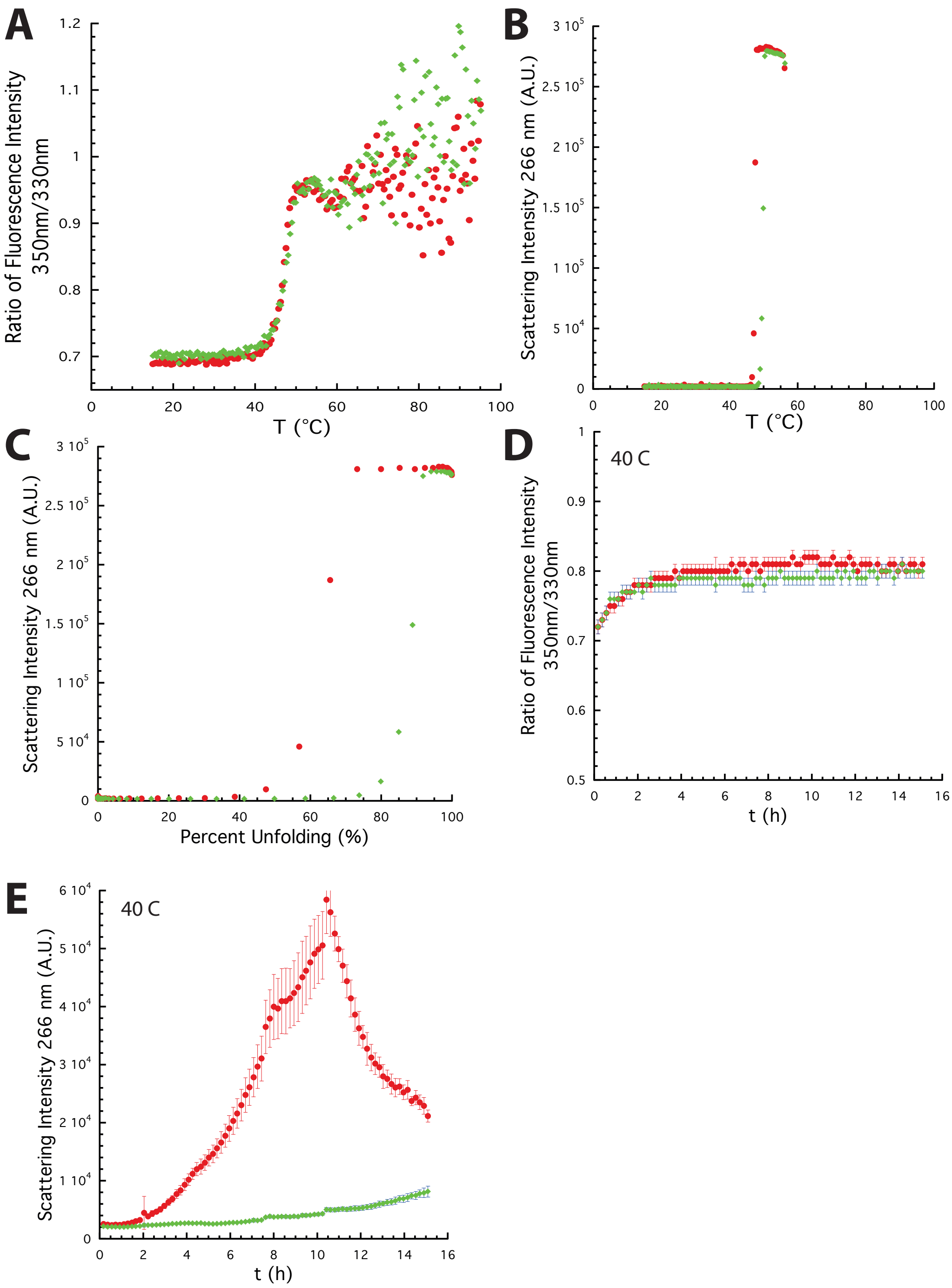

Supplementary Figure 5 – Aggregation kinetics of wild type and mutant Protective Antigen. (A) Intrinsic tryptophane fluorescence monitored as the ratio of emission at 350 by 330 nm for a temperature ramp experiment of wild type (red) and S559L/T576E (green) Protective Antigen. **(B)** Static Light scatterig of the same temperature ramp experiment as shown in panel A. **(C)** The same scattering data as in panel B but plotted against the degree of unfolding as the x-axis. **(D)** Companion to Figure 7F, showing the evolution of the intrinsic tryptophane fluorenscence emission ratio at 350 and 330 nm over time at 40 °C. Compare to panel A for full range of unfolding at higher temperatures. **(E)** Companion to Figure 7F, showing the static light scattering intensity at 266 nm.

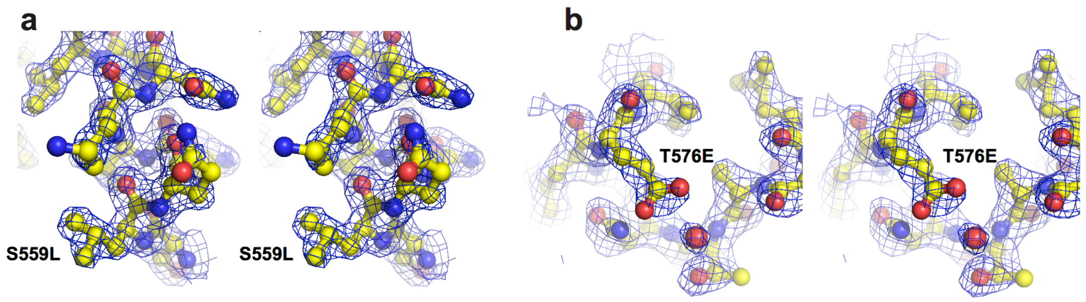

Supplementary Figure 6 – Stereo image of the electron density shown in main figure 8 C (a) and 8 D (b).

Supplementary Table

Supplementary Table 1 – Crystallographic parameters for 5fr3.

	APA S559L/T576E
Crystallographic statistics	
Beamline	X06A (SLS)
Date of collection	13-Feb-13
Wavelength (Å)	0.92045
Spacegroup	$P2_12_12_1$
a,*b*,*c* (Å)	84.59, 94.73, 100.68
α,β,γ (°)	90.0, 90.0, 90.0
Resolution limits (Å)	44.45 - 1.94 (2.04 - 1.94)
R_{merge} (%)	11.2 (83.4)
R_{meas} (%)	13.2 (98.9)
$\langle I/\sigma \rangle$	8.0 (1.4)
$CC_{1/2}$ (%)	99.5 (58.5)
Multiplicity	3.4 (3.2)
Completeness (%)	98.7 (97.2)
Total number of reflections	203741 (27308)
Number unique reflections	59668 (8478)
Refinement and model statistics	
R_{work} (%)	17.8
R_{free} (%)	22.7
Rmsd bond distance (Å)	0.005
Rmsd bond angle (°)	0.953
Ramachandran outliers	0
Ramachandran favored (%)	97.4
MolProbity score (%)	96

Supplementary Reference

1. Niwa T, *et al.* Bimodal protein solubility distribution revealed by an aggregation analysis of the entire ensemble of Escherichia coli proteins. *Proceedings of the National Academy of Sciences of the United States of America* **106**, 4201-4206 (2009).
